# The age- and sex-specific decline of the 20s proteasome and the Nrf2/CncC signal transduction pathway in adaption and resistance to oxidative stress in *Drosophila melanogaster*

**DOI:** 10.18632/aging.101218

**Published:** 2017-04-03

**Authors:** Laura C.D. Pomatto, Sarah Wong, Caroline Carney, Brenda Shen, John Tower, Kelvin J. A. Davies

**Affiliations:** ^1^ Ethel Percy Andrus Gerontology Center, Leonard Davis School of Gerontology, University of Southern California, Los Angeles, CA 90089, USA; ^2^ Molecular and Computational Biology Program, Department of Biological Sciences, Dornsife College of Letters, Arts and Sciences, University of Southern California, Los Angeles, CA 90089, USA

**Keywords:** 20S proteasome, protein aggregation, Nrf2, adaptive homeostasis, oxidative stress, protein oxidation

## Abstract

Hallmarks of aging include loss of protein homeostasis and dysregulation of stress-adaptive pathways. Loss of adaptive homeostasis, increases accumulation of DNA, protein, and lipid damage. During acute stress, the Cnc-C (*Drosophila* Nrf2 orthologue) transcriptionally-regulated 20S proteasome degrades damaged proteins in an ATP-independent manner. Exposure to very low, non-toxic, signaling concentrations of the redox-signaling agent hydrogen peroxide (H_2_O_2_) cause adaptive increases in the *de novo* expression and proteolytic activity/capacity of the 20S proteasome in female *D. melanogaster* (fruit-flies). Female 20S proteasome induction was accompanied by increased tolerance to a subsequent normally toxic but sub-lethal amount of H_2_O_2_, and blocking adaptive increases in proteasome expression also prevented full adaptation. We find, however, that this adaptive response is both sex- and age-dependent. Both increased proteasome expression and activity, and increased oxidative-stress resistance, in female flies, were lost with age. In contrast, male flies exhibited no H_2_O_2_ adaptation, irrespective of age. Furthermore, aging caused a generalized increase in basal 20S proteasome expression, but proteolytic activity and adaptation were both compromised. Finally, continual knockdown of Keep1 (the cytosolic inhibitor of Cnc-C) in adults resulted in older flies with greater stress resistance than their age-matched controls, but who still exhibited an age-associated loss of adaptive homeostasis.

## INTRODUCTION

Multiple byproducts of cellular metabolism, with which aerobic organisms must cope, include numerous free radicals and reactive oxygen/nitrogen species, which can damage lipids, proteins, and DNA [1]. If damaged proteins are not immediately removed, hydrophobic protein aggregates can accumulate, cross-link, and accelerate cellular senescence [2, 3]. To cope with these insults, cells rely upon an array of stress responsive enzymes, including the well-characterized 20S proteasome, which can rapidly degrade oxidized proteins in the cytoplasm, nucleus and endoplasmic reticulum, thus preventing proteins from forming toxic cross-linked aggregates [4-6]. The Lon protease performs the same protective function inside mitochondria which do not have proteasomes [7].

Transient short-term adaptation, or ‘adaptive homeostasis’ is a widely-characterized phenomenon that can protect against damage accrual from environmental or physiological stresses [8]. The adaptive homeostasis process describes the ability of cells, tissues, or organisms, to activate various stress-responsive pathways, including *de novo* synthesis of the 20S proteasome, in response to exposure to very low and non-toxic levels of a stimulating agent or condition. Protective enzymes synthesized during adaptive homeostasis then act as a means to mitigate against future oxidative insult, even levels of toxicants that might otherwise be severely damaging or lethal [9, 10]. The response is not binary, but rather exhibits a dynamic range, that enables the fine-tuning in its activation. With age, this dynamic range of adaptive responses compresses [11, 12]. As a result, the ability to adapt to varying levels of oxidative stress declines.

Accumulation of oxidized proteins is a hallmark of aging [2, 3], and is indicative of a decline in protein turnover [13]. Conversely, long-lived organisms, including human centenarians, maintain their homeo-static balance between protein degradation and turnover [14-16]. The loss of proteostasis has largely been attributed to the dysregulation of the ubiquitin-proteasome system (UPS), assessed by the degradation of ubiquitin-tagged proteins by the 26S proteasome, which is comprised of the 20S catalytic core and 19S regulatory caps on each end [17, 18]. Indeed, age-related aggregation of polyubiquintated proteins is evident in studies ranging from mammalian cell cultures to humans [19-21].

However, polyubiquitaition is not the only means for protein turnover, as oxidized proteins have been shown to be degraded, independent of ubiquitin tagging [22-25]. Furthermore, activity of the ubiquitin activating/conjugating system, the main signal for protein degradation by the 26S proteasome, is actually suppressed during oxidative stress [26]. In addition, the 26S proteasome undergoes transient disassembly, (into free 20S proteasomes and 19S regulators bound to HSP70) in a process catalyzed by HSP70 and Ecm29 [27, 28]. The release of ATP-independent free 20S proteasomes, many of which immediately attach to 11S (also called Pa28) regulators, ensures immediate degradation of oxidized proteins [23, 27]. Studies in mouse models found aging, alone, does not accelerate protein ubiquitinylation, further weakening the age-related importance of the 26S proteasome which is the primary means of turning over ubiquitin tagged proteins [29]. Nor do the 19S regulatory caps appear essential, as oxidative stress can render them inactive, irrespective of age [30], and deletion of the 19S caps is not lethal [31]. Taken together, these findings indicate the need to reassess the predominant focus given to the ubiquitin-proteasome system as the primary marker for age-associated declines in protein turnover.

Much of the work on aging and proteostasis has been undertaken in male animal models, yet it is becoming abundantly clear that there are significant differences in male and female patterns of aging. Moreover, the fruit fly *D. melanogaster* offers excellent opportunities to explore differences in both basal stress resistance, and adaptive stress responses between the sexes at all ages. Sexual differences, or sexual dimorphism, is partly a consequence of the maternal transmission of the mitochondrial genome [32-34]. Indeed, it has been suggested that the asymmetry of mitochondrial inheritance may result in differences in lifespan (typically favoring females) as evident in flies [34-37], mice [38, 39], and humans [40]. Moreover, females typically show higher levels of stress resistance [34, 37, 38, 41, 42]. As well, more recent studies have shown that the adaptive stress response is inducible in a female-specific manner [43, 44]. Therefore, the central tenant of this study was to further understand the age-associated changes, between the sexes, in regards to the basal levels and inducibility of the 20S proteasome.

To us, this work highlights the importance of assessing the age-associated and sex-specific decline of the adaptive response of the ATP-independent 20S proteasome catalytic core. The model organism, *D. melanogaster*, was utilized in order to assess the age-related and sex-specific differences in the adaptive response of the 20S proteasome.

Three day (Young) and 60 day old (Aged) flies were pretreated with adaptive (signaling) concentrations of hydrogen peroxide (10μM and 100μM H_2_O_2_) to assess the inducibility of the 20S proteasome (both expression and activity). Following pretreatment, flies were subjected to toxic, oxidative stress-inducing con-centrations of H_2_O_2_ to determine changes in survival (adaptation). The impact of suppressing the adaptive increase of the 20S proteasomal beta subunits upon stress survival and lifespan was also explored. Lastly, in an attempt to restore the adaptive response in aged animals, flies were subjected to a chronic knock-down of Keap1, the cytosolic inhibitor of Cnc-C/Nrf2, prior to assessing changes in stress survival.

## RESULTS

### Hydrogen peroxide resistance is diminished with age

Prior studies suggest an age-related decline in stress resistance of *D. melanogaster* to stresses such as starvation [45] and the redox-cycling agent paraquat [46], resulting in increased mortality. The present study sought to compare the age-associated changes in survival of both male and female flies following exposure to toxic levels of hydrogen peroxide (H_2_O_2_). A prior study found an increase in H_2_O_2_ sensitivity within 35 day old flies [47], arguably ‘middle-age’ in typical control strains of *D. melanogaster* [48]. Here, we sought to extend this, by comparing sensitivity in 3 day and 60 day old flies. 3 day old flies were selected to represent young [43, 44], and 60 day old flies were selected to represent aged animals [49]. The selection of 60 day old flies was influenced by the fact that more than 80% of the population was still alive at this point. Had an even older population been chosen to study, these results might have suffered from a selection bias in favor of the oldest survivors.

**Figure 1:**
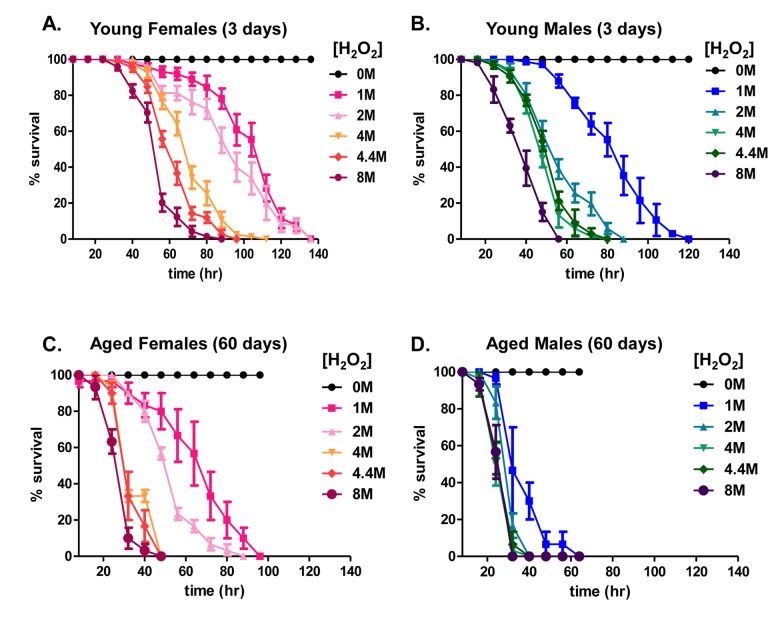
Hydrogen peroxide resistance declines with age (**A-D**) Males and females of the Actin-GS-255B strain crossed to the w[1118] strain were aged to 3 days or 60 days and then transferred to vials containing kimwipes soaked in 1.0M to 8.0M H_2_O_2_ dissolved in 5% sucrose solution, and flies were scored as dead when completely immobile, as described previously [43]. (**A**) 3 day old females. (**B**) 3 day old males. (**C**) 60 day old females. (**D**) 60 day old males.

Flies were provided food (5% sucrose) without H_2_O_2_, or with the addition of H_2_O_2_ at concentrations from 1.0M to 8.0M, on Kimwipes as previously described [43]. Survival was scored every 8 hours. High concentrations of H_2_O_2_ caused roughly similar toxicity in both young males and females (Fig. 1A and 1B). With age, however, males and females became more sensitive to H_2_O_2_ toxicity, with survival decreasing by more than 30 hours at the lowest concentration in females, and by more than 60 hours in males (Fig. 1C and 1D). Thus, females appear to be significantly more stress-resistant with age than do males.

### Hydrogen peroxide adaptation is specific to females and is lost with age

**Figure 2:**
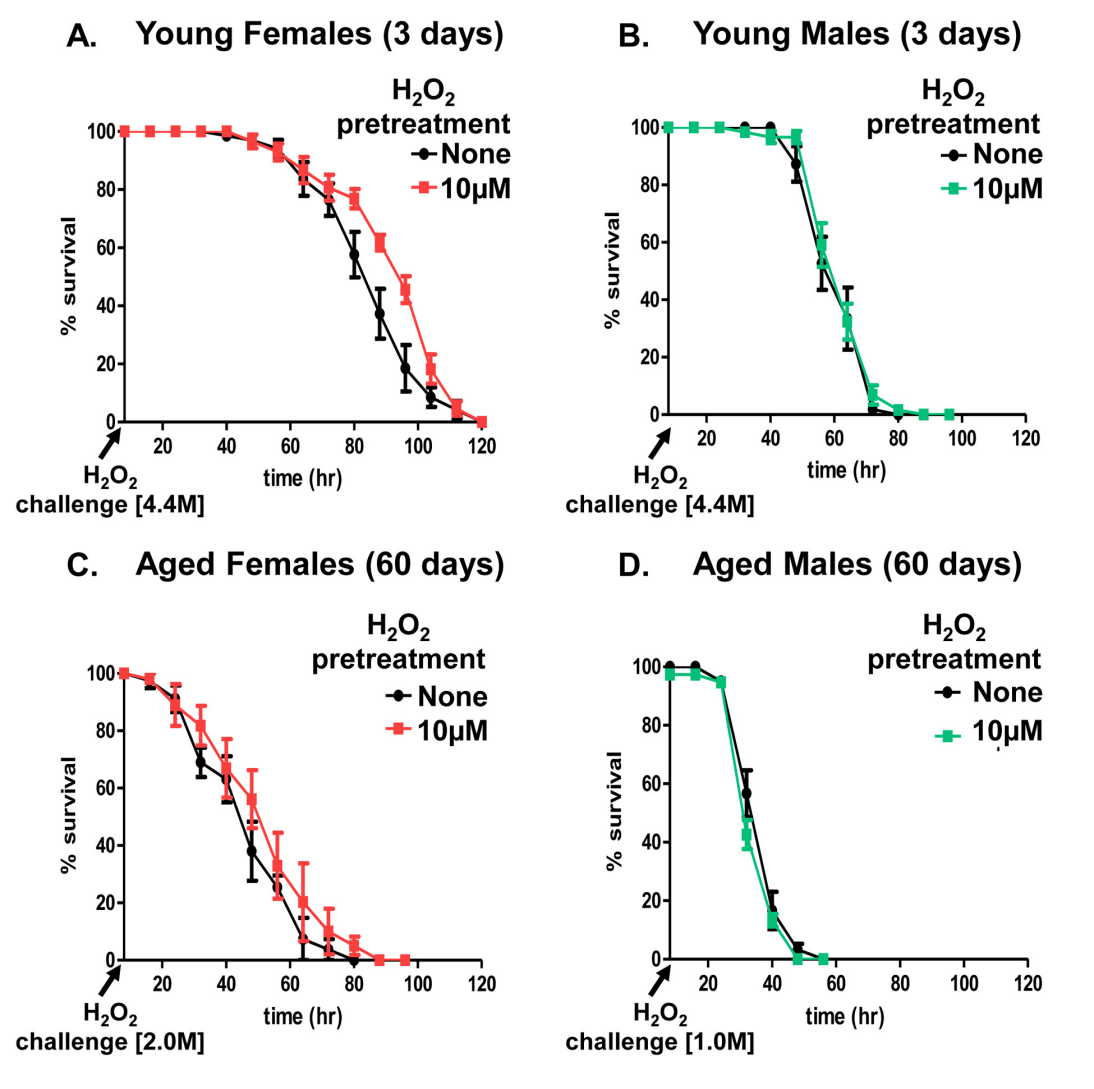
Adaptation to hydrogen peroxide declines with age in females Progeny of the Actin-GS-255B strain crossed to the w[1118] strain were aged to 3 days and 60 days prior to H_2_O_2_ pretreatment. Following recovery, flies were fed H_2_O_2_ challenge dose: 4.4M in 3 day old males and females, 2M in 60 day old females, and 1M in 60 day old males. (**A**) 3 day old females showed increased survival following pretreatment. (**B**) 3 day old males showed no change in survival following pretreatment. (**C**) With age, 60 day old females no longer show increased survival. (**D**) 60 day old males show no change in adaptation following pretreatment. Statistical difference in survival (p < 0.05) was calculated using the Log-Rank test. Statistical summary is located in Supplementary Table S1.

Adaptive homeostasis [8] is the transient physiological activation of the cellular stress response pathways, which allow cells [50, 51], tissues [52], and whole organisms [43, 44, 53] to withstand future oxidative insult for a period of time; the effectiveness of adaptive homeostasis diminishes with age. A prior study assessing H_2_O_2_-mediated adaptation, found 35-day old (‘middle-age’) females were no longer able to adapt [47]. Here, we sought to expand these findings and test the age-related changes in adaptation between 3 day (young) and 60 day (old) flies. Flies from both sexes and age groups were either not pretreated with H_2_O_2_, used as controls, or flies were pretreated with 10μM H_2_O_2_ for 8 hours, and then allowed a further 16 hours to adapt, prior to exposure to a toxic dose of H_2_O_2_. Previous studies had confirmed similar uptake and ingestion of H_2_O_2_ by both males and females over the 8-hour period [43]. Young females, showed increased survival upon exposure to the 4.4M toxic dose of H_2_O_2_ if they were first pre-treated with the 10μM adaptive dose of H_2_O_2_ (Fig. 2A and Supplementary Table S1). In contrast, aged females no longer exhibited increased survival after pretreatment (Fig. 2C and Supplementary Table S1). Remarkably males, regardless of age or pretreatment, displayed no adaptive response to H_2_O_2_ (Fig. 2B, 2D, and Supplementary Table S1). Moreover this finding built upon a previous result from our lab, which also demonstrated lack of adaptation explored only in young males [43]. It should be noted that, since aged flies were no longer capable of withstanding the same 4.4M H_2_O_2_ toxic dose used with young flies (Fig. 1A, 1B, 1C, and 1D), 2.0M H_2_O_2_ was used as the toxic dose in aged females and 1.0M H_2_O_2_ as the toxic dose in males.

### Adaptive *de novo* expression of the 20S proteasome diminishes with age in a sex-dependent manner

**Figure 3:**
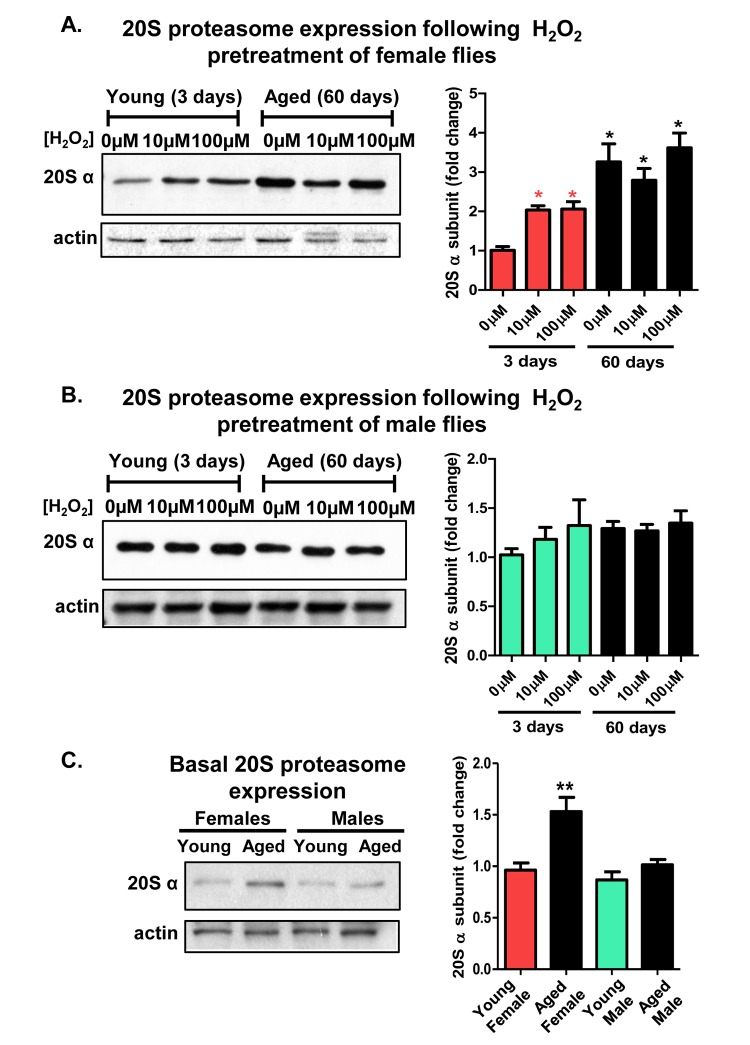
Adaptive de novo expression of the 20S proteasome diminishes with age in a sex-dependent manner (**A,B**) Virgin females of the Actin-GS-255B strain were crossed to males of the w[1118] strain and progeny were assayed for 20Sα expression without H_2_O_2_ pretreatment, or after pretreatment with [10μM or 100μM] H_2_O_2_. (**A**) 3 day and 60 day aged females. (**B**) 3 day and 60 day aged males. All samples were compared to the 3 day old 0μM H_2_O_2_ controls (**C**) Basal expression of the 20S proteasome α subunits was measured between 3 day old females, 60 day old females, 3 day old males, and 60 day old males, with samples normalized to the 3 day old female. Western blots were performed in triplicate, normalized to Actin-HRP, and quantified using ImageJ. Error bars denote standard error of the mean (S.E.M) values. * *P* <0.05 and ** *P* < 0.01, relative to control using one-way ANOVA. Asterisks color indicates the age of the sample, young female (pink *) and aged female (black *). All statistical significance was calculated relative to the young controls.

Prior studies of mammalian cell cultures have demonstrated that the 20S proteasome is inducible, following oxidative stress [23, 51, 54], but proteolytic capacity diminishes with nominal age or senescence [55]. To understand the age-related changes in the adaptive expression of the 20S proteasome, 3 day old and 60 day old male and female fruit flies were studied. Following H_2_O_2_ [0μM-100μM] pretreatment, entire flies were homogenized and the lysates were analyzed by western blot. The blots were incubated with an antibody directed against the *D. melanogaster* α-subunit of the 20S proteasome, and protein loading was normalized to actin. Total lysates from young females showed robust increase in proteasome expression, however, aged females exhibited no H_2_O_2_ inducible increase in proteasome levels (Fig. 3A). Importantly, although they were no longer capable of inducing 20S proteasome expression, basal proteasome levels in aged females actually matched, or exceeded, the maximum H_2_O_2_ inducible induction achieved by young females (Fig. 3A). In contrast, total lysates from males showed no change in 20S proteasome expression regardless age or pretreatment (Fig. 3B). To be able to directly compare both age-related and sex-specific differences in basal proteasome expression, young and old female and male lysate were next analyzed by western blot, under identical conditions in a single experiment. Interestingly, aged females reproducibly demonstrated a higher basal proteasome expression compared to young females, whereas, males showed no difference with age (Fig. 3C).

### Adaptive proteolytic capacity of the 20S proteasome diminishes with age in a sex-dependent manner

**Figure 4:**
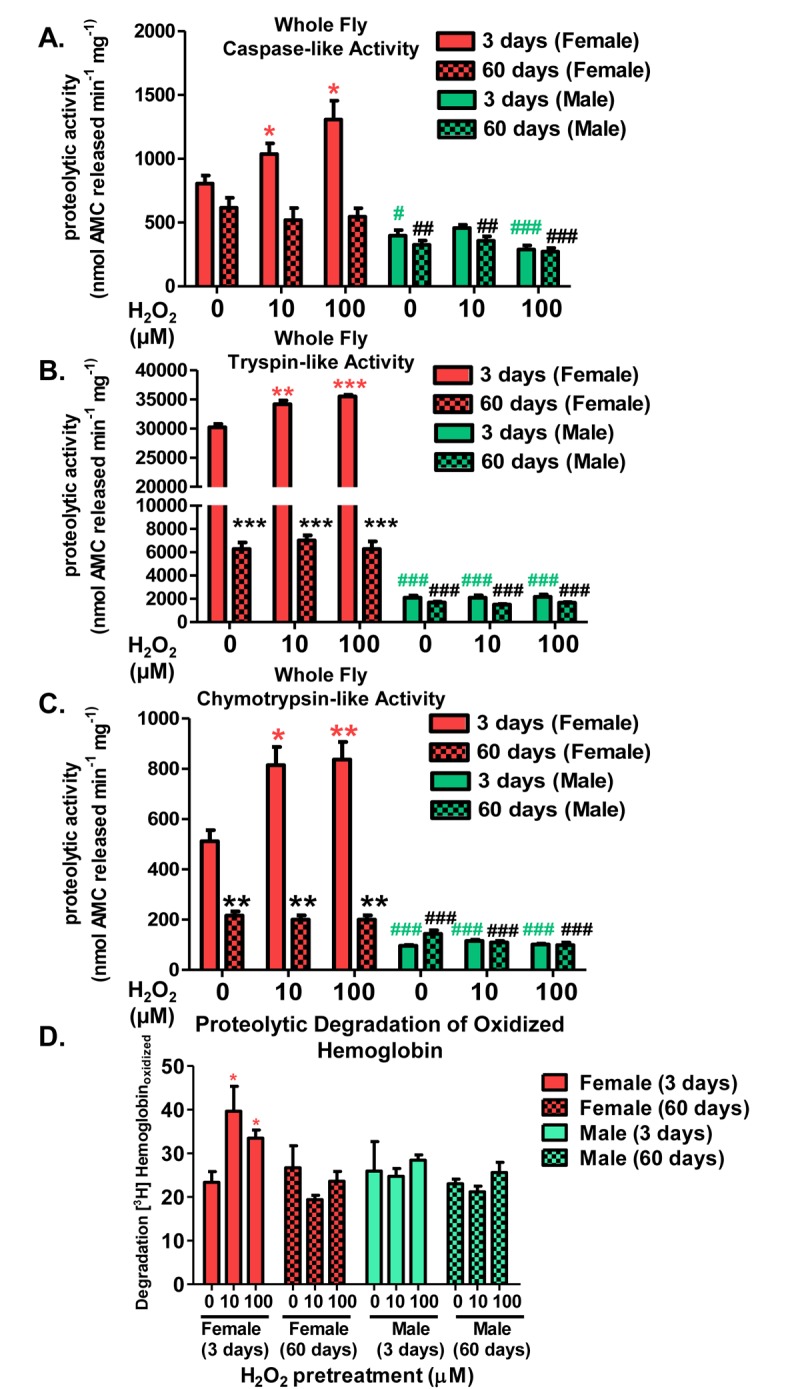
Adaptive proteolytic capacity of the 20S proteasome diminishes with age in a sex-dependent manner Virgin females of the Actin-GS-255B strain were crossed to males of the w[1118] strain and progeny were assayed for the proteolytic activity of the three catalytic subunits of the 20S proteasome in 3 day-old (red or blue) and 60 day-old flies (checked pattern). Caspase-like activity in (**A**) Females and males. Trypsin-like activity in (**B**) Females and males. Chymotrypsin-like activity in (**C**) Females and males. (**D**) Proteolytic degradation of oxidized [^3^H] hemoglobin in flies pretreated with hydrogen peroxide at 3 days and 60 days. Statistical significance for proteolysis of oxidized substrate was compared to young control females. Error bars denote standard error of the mean (S.E.M) values. * *P* <0.05, ** *P* < 0.01, *** *P* < 0.001 relative to control using one-way ANOVA. Asterisk color corresponds to young females (pink *), aged females (black *), young males (green #), and aged males (black #). Statistical significance was calculated relative to the young control females (**A-D**).

Because proteasome expression was inducible in a sex-specific manner, proteolytic activity of the 20S proteasome was also measured. 3 day old and 60 day old males and females were either not pretreated with H_2_O_2_ (controls), or were pretreated for 8 hours, with either 10μM or 100μM H_2_O_2_ as adaptive doses, then allowed a further 16-hour interval to adapt, before sample collection commenced. Proteolytic capacity was measured in total lysates by the ability to degrade model fluorogenic peptides, designed to be specific for each of the proteasome's three proteolytic activities: Z-LLE-AMC for caspase-like/β1 activity, Z-ARR-AMC for trypsin-like/β2 activity, and Suc-LLVY-AMC for chymotrypsin-like/β5 activity. After exposure to increasing concentrations of H_2_O_2_, young females (pink) showed a marked increase in proteolytic capacity in all three proteasomal subunits (Fig. 4). In contrast, aged females (pink checkered), showed loss of protea-some induction and basal decline in proteasome degradation (Fig. 4A, 4B, and 4C). Increased proteolytic capacity was shown to be proteasome-dependent, as inhibition of proteolytic activity by the proteasome selective inhibitors lactacystin and epoxomycin blocked the inductive response evident in young pretreated females (Supplementary Fig. S1A and S1B). Both, young (green) and aged males (green checkered) showed no increase in proteolytic capacity following H_2_O_2_ pretreatment, nor a basal change in proteolytic capacity with age (Fig. 4A, 4B, and 4C). As oxidized proteins are the primary substrates of the 20S proteasome [5, 6], lysate from whole flies were incubated with ([^3^H]-labeled) oxidized hemoglobin ([^3^H]Hb_oxdized_) and proteolysis was assessed by increased acid-soluble, low molecular-weight, scintil-lation counts (from the originally acid-precipitable small ^3^H-peptides and ^3^H-amino acids released by proteolysis). Young females, pretreated with H_2_O_2_, showed increased proteolytic degradation compared to controls, whereas with age, H_2_O_2_-induced proteolytic capacity was lost. Males, irrespective of pretreatment or age, showed no change in proteolysis following pretreatment (Fig. 4D).

### The adaptive expression of the 20S proteasome is age and tissue-dependent in females

**Figure 5:**
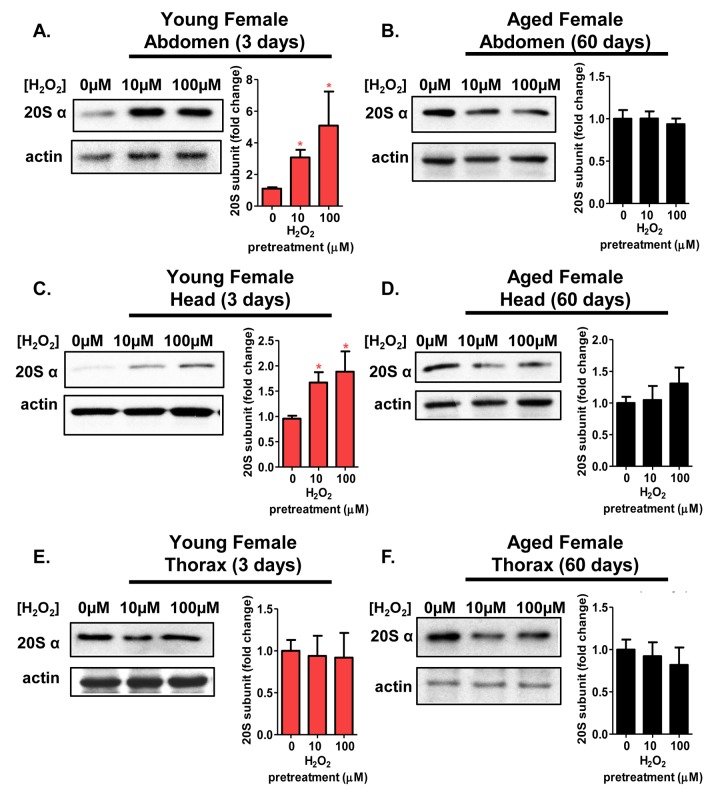
The adaptive expression of the 20S proteasome is age and tissue-dependent in females Body segments collected from females of the Actin-GS-255B strain crossed to the w[1118] strain were used as controls, or were pretreated with either 10μM or 100μM hydrogen peroxide. (**A,B**) 20Sα expression in female abdominal tissue following pretreatment. (**A**) 3 day old. (**B**) 60 day old. (**C,D**) 20Sα expression in female head following pretreatment. (**C**) 3 day old. (**D**) 60 day old. (**E,F**) 20Sα expression in female thorax following pretreatment. (**E**) 3 day old. (**F**) 60 day old. Western blots were performed in triplicate, normalized to Actin-HRP, and quantified using ImageJ. The bar charts represent the quantification. Error bars denote standard error of the mean (S.E.M) values. * *P* <0.05, ** *P* <0.01, *** *P* < 0.001, relative to the female control using one-way ANOVA. Statistical significance is indicated in young females with pink asterisks (pink *).

Next, it was important to determine whether various tissues displayed differences in the adaptive response of the 20S proteasome. 3 day old and 60 day old flies were either used as controls, or were pretreated with 10μM or 100μM H_2_O_2_ before body segments (head, thorax, and abdomen) were collected. Expression of the 20S proteasome was assessed in tissue homogenate by western blot, as in Figure 3. Young females showed a marked increase in 20S proteasome expression within the abdomen and the head (Fig. 5A and 5C). However, thorax from young females showed no induction of the 20S proteasome, regardless of pretreatment, (Fig. 5E). With age, the adaptive response visible in the 3 day old abdomen and head were lost (Fig. 5B and 5D), and there was again no change in 20S proteasome expression after pretreatment in thoraxes from 60 day old females (Fig. 5F).

**Figure 6:**
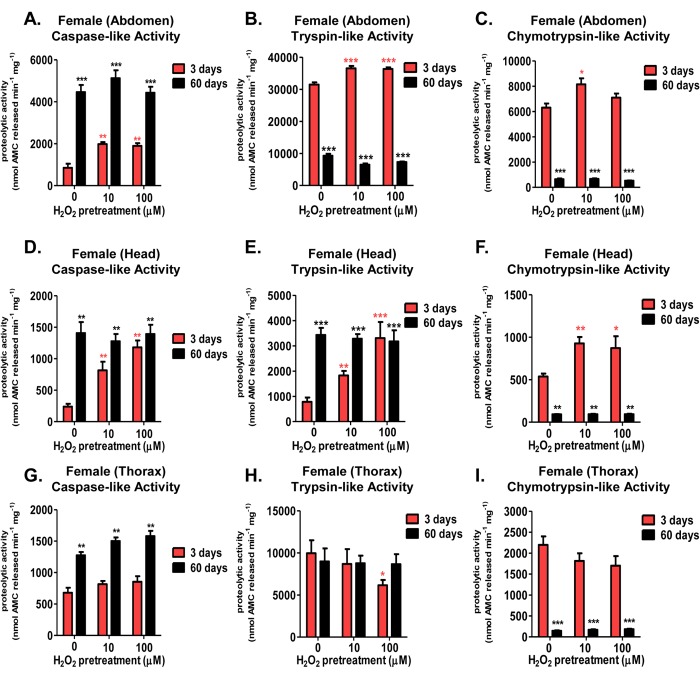
Tissue-specific differences of the adaptive proteolytic capacity, and age-dependent changes in basal activity, of the 20S proteasome in females Body segments collected from female progeny of the Actin-GS-255B strain crossed to the w[1118] strain were used as controls, or were pretreated with either 10μM or 100μM hydrogen peroxide. Individual proteolytic capacity of the 20S proteasome (caspase/peptidyl glutamyl-peptide hydrolyzing-like activity, trypsin-like, and chymotrypsin-like activity) was measured in the abdomen, head, and thorax. (**A-C**) Abdomen isolated from 3 day old (pink) and 60 day old (black) females following hydrogen peroxide pretreatment. (**A**) Caspase-like activity. (**B**) Trypsin-like activity. (**C**) Chymotrypsin-like activity. (**D-F**) Head isolated from 3 day old (pink) and 60 day old (black) females following hydrogen peroxide pretreatment. (**D**) Caspase-like activity. (**E**) Trypsin-like activity. (**F**) Chymotrypsin-like activity. (**G-I**) Thorax isolated from 3 day old (pink) and 60 day old (black) females following hydrogen peroxide pretreatment. (**G**) Caspase-like activity. (**H**) Trypsin-like activity. (**I**) Chymotrypsin-like activity. Error bars indicate the standard error of the mean (S.E.M) values. * *P* <0.05, ** *P* <0.01, *** *P* < 0.001, relative to the young female control using one-way ANOVA. Statistical significance is shown with pink asterisks (pink *) for young females and black asterisks (black *) in aged females.

The proteasome core is crucial to the rapid removal of damaged proteins from cells and tissues. However, loss of protein turnover has been considered to be a hallmark of aging [55, 56], accompanied by diminished basal proteolytic capacity of the 20S proteasome [57, 58], though this finding is somewhat controversial [20, 53, 59-61]. To explore this matter further, tissue lysate (abdomen, head, thorax) from 3 day old and 60 day old females, pretreated with various concentrations of H_2_O_2_ [0μM-100μM] were assessed for changes in proteolytic capacity (measured fluorometrically) following the addition of fluorogenic peptides specific for the three proteolytic activities of the 20S proteasome core. Similar to the increased proteasome expression (seen in Fig. 5), all three proteasomal proteolytic activities in the abdomen and head exhibited significant increases in 3 day old H_2_O_2_ pretreated females (Fig. 6A, 6B, 6C, 6D, 6E, and 6F). No such adaptive increase in proteolytic capacities was observed, however, in 60 day old females (Fig. 6A, 6B, 6C, 6D, 6E, 6F). Thoracic tissue showed no change in proteolysis, regardless of age or pretreatment (Fig. 6G, 6H, and 6I). Thus the results of Figures 5 and 6 demonstrate an excellent correlation between the loss of proteasome protein inducibility (Fig. 5) and diminished ability to increase actual proteasome proteolytic activity (Fig. 6) with age and tissue type.

### Males show no tissue-specific or age-related adaptation of the 20S proteasome

**Figure 7:**
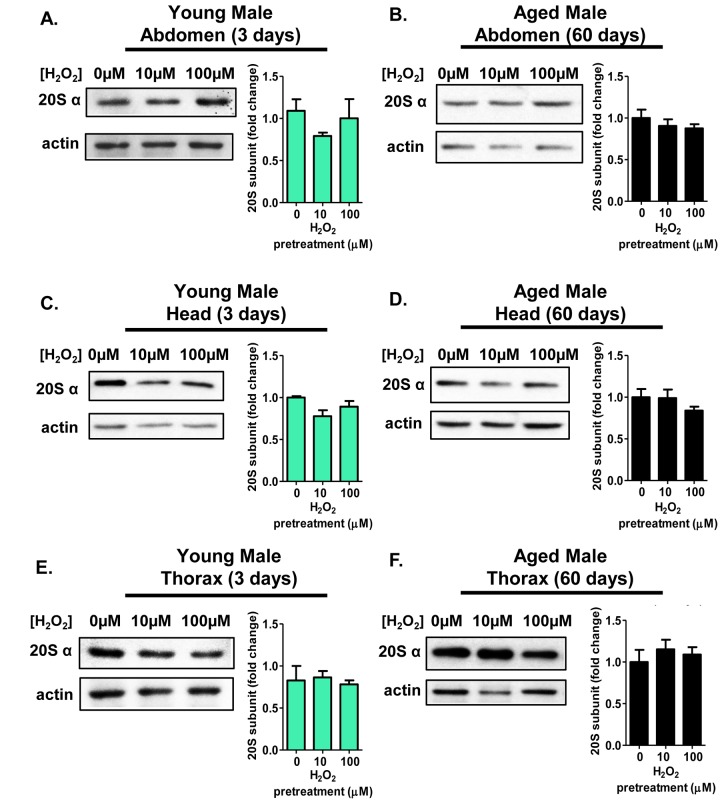
Males show no tissue-specific or age-related adaptive changes in 20S proteasome protein levels Body segments were collected from males of the Actin-GS-255B strain crossed to the w[1118] strain that were used as controls, or that were pretreated with either 10μM or 100μM hydrogen peroxide. (**A,B**) 20Sα expression in male abdominal tissue following pretreatment. (**A**) 3 day old. (**B**) 60 day old. (**C,D**) 20Sα expression in male head following pretreatment. (**C**) 3 day old. (**D**) 60 day old. (**E,F**) 20Sα expression in male thorax following pretreatment. (**E**) 3 day old. (**F**) 60 day old. Western blots were performed in triplicate, normalized to Actin-HRP, and quantified using ImageJ. The bar charts represent the quantification. Error bars denote standard error of the mean (S.E.M) values, relative to the male control using one-way ANOVA.

Whole tissue extracts showed no change in proteasome expression or activity in males following any H_2_O_2_ pretreatment tested. It should also be noted that it was tested at several higher concentrations in males, none of which caused adaptation (data not shown) [43]. Although no proteasome induction was observed in whole tissue extracts from males, we sought to explore if any differences existed within the three major body segments (head, thorax, and abdomen) in *D. melanogaster* males, because male body segments are not equal in size are not equal in size. Therefore, it was important to test for possible proteasome changes in the three major body segments separately. For these experiments, 3 day old and 60 day old males were pretreated with varying amounts of H_2_O_2_ [0μM-100μM], and allowed an opportunity to adapt for an additional 16 hours, prior to body segment collection (head, abdomen, and thorax). Following pretreatment, 20S expression in the abdomen, head, and thorax remained unchanged, irrespective of the pretreatment regimen used (Fig. 7A, 7C, and 7E) or age (Fig. 7B, 7D, and 7F). The lack of body segment-specific adaptive responses in males recapitulates the earlier finding of whole male lysate showing no adaptive response to H_2_O_2_ pretreatment [43].

**Figure 8:**
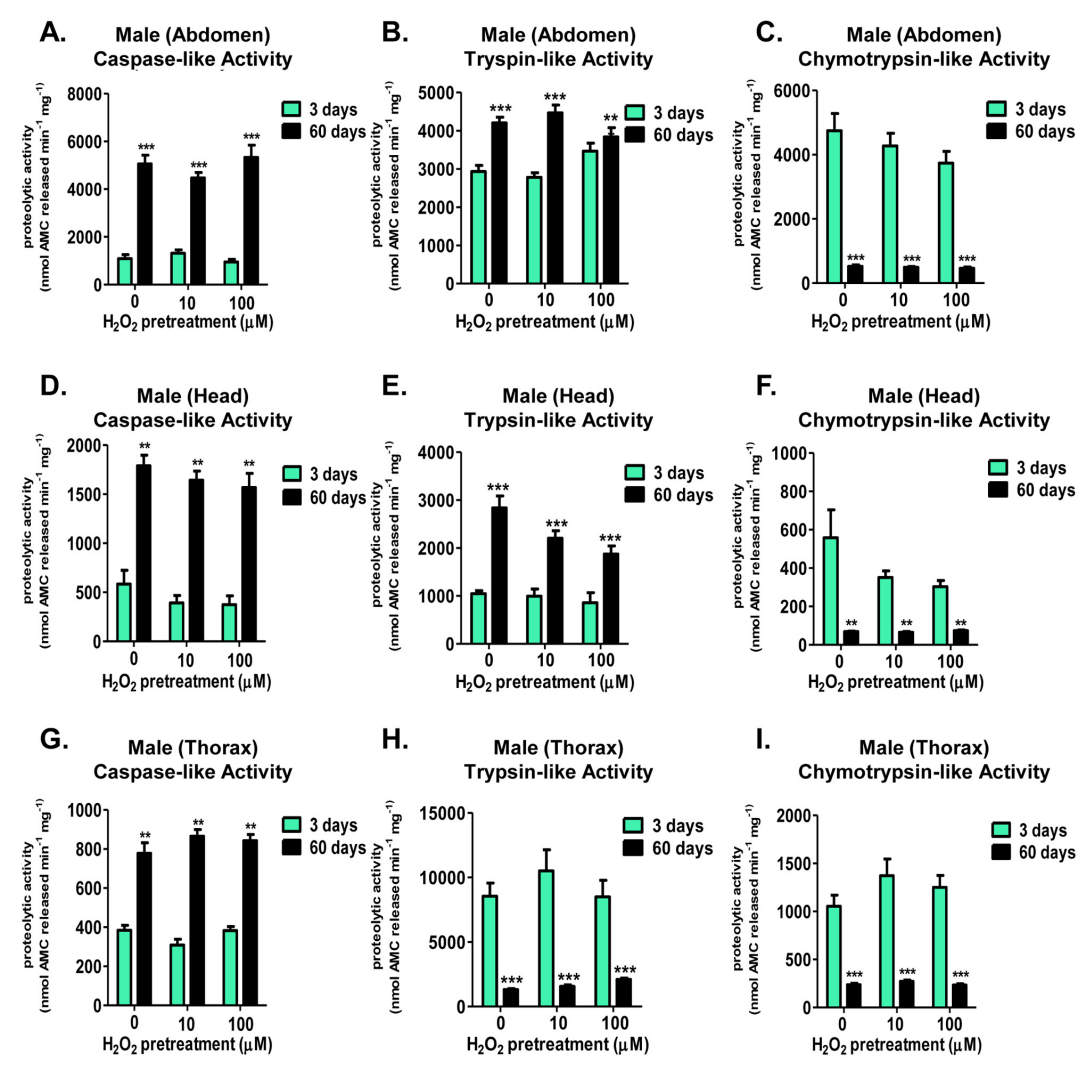
Males show no tissue-specific differences in the adaptive proteolytic capacity of the 20S proteasome, but do exhibit age-dependent changes in proteasomal basal activity Body segments were collected from male progeny of the Actin-GS-255B strain crossed to the w[1118] strain that were used as controls, or that were pretreated with either 10μM or 100μM hydrogen peroxide. Individual proteolytic capacity of the 20S proteasome (caspase/peptidyl glutamyl-peptide hydrolyzing-like activity, trypsin-like, and chymotrypsin-like activity) was measured in the abdomen, head, and thorax. (**A-C**) Abdomen isolated from 3 day old (green) and 60 day old (black) males following hydrogen peroxide pretreatment. (**A**) Caspase-like activity. (**B**) Trypsin-like activity. (**C**) Chymotrypsin-like activity. (**D-F**) Head isolated from 3 day old (green) and 60 day old (black) males following hydrogen peroxide pretreatment. (**D**) Caspase-like activity. (**E**) Trypsin-like activity. (**F**) Chymotrypsin-like activity. (**G-I**) Thorax isolated from 3 day old (green) and 60 day old (black) males following hydrogen peroxide pretreatment. (**G**) Caspase-like activity. (**H**) Trypsin-like activity. (**I**) Chymotrypsin-like activity.

Next, proteolysis was assessed within the young and aged pretreated male tissues (head, abdomen, and thorax). Pretreated lysates were incubated in the presence of fluorogenic substrates, specific for each of the proteasome's caspase-, trypsin-, and chymotrypsin-like activities. Irrespective of which body segment was studied, there were no adaptive changes in caspase-like activity (Fig. 8A, 8D, and 8G), trypsin-like activity (Fig. 8B, 8E, and 8H), or chymotrypsin-like activity, following H_2_O_2_ pretreatment (Fig. 8C, 8F, and 8I).

### Age-dependent changes occur in the basal activities of the 20S proteasome in both females and males

Interestingly, both females and males exhibited marked age-dependent changes in basal (non-induced) levels of the three proteasomal activities within the three body segments tested. Specifically, aged females showed basal increases in the caspase-like activity in all three tissues (Fig. 6A, 6D, and 6G), and no significant change in the trypsin-like activity within the abdomen or thorax (Fig. 6E and 6H). In contrast, aged females showed a robust decrease in the chymotrypsin-like activity in all tissues (Fig. 6C, 6F, and 6I). Similarly, basal caspase-like activity showed a robust increase with age within all male tissues (Fig. 8A, 8D, and 8G). As well, male head and abdominal tissue showed marked increases in basal trypsin-like activity with age (Fig. 8B and 8E).

And, similar to females, basal chymotrypsin-like activity showed a significant decrease in aged maletissue (Fig. 8C, 8F, and 8I). This dramatic difference between the individual activities of the 20S catalytic core with age (basal elevation in the caspase- and trypsin-like, whereas a dramatic decline in the chymotrypsin-like activity) may hint at the underlying decrease in protein degradation, regardless of the increase in basal protein expression [20]. Furthermore, this finding may point to proteasome's chymotrypsin-like activity being the potential rate-limiting step in protein degradation by the 20S core.

### Adaptation is dependent upon the 20S proteasome

**Figure 9:**
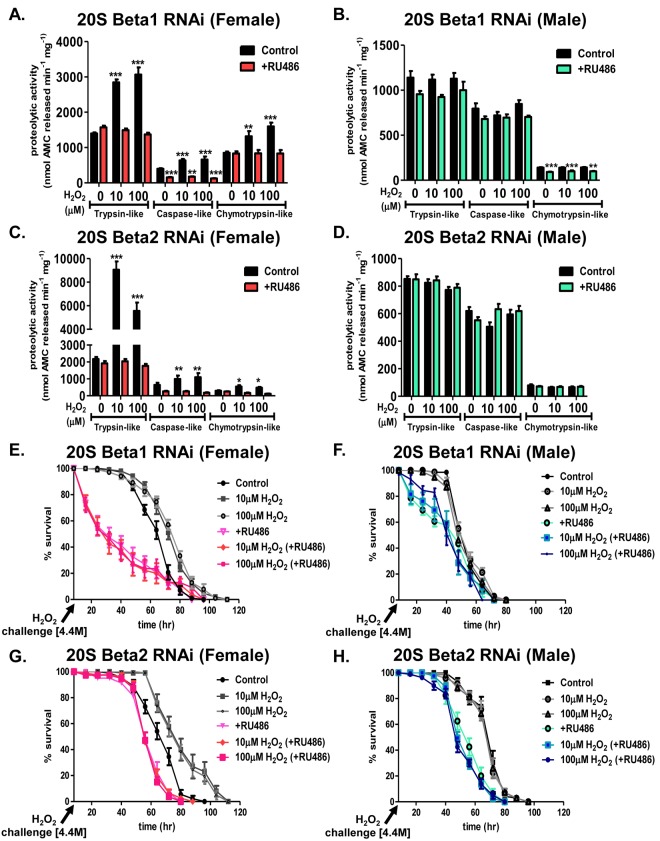
Adaptation is dependent upon the 20S proteasome Progeny of the Actin-GS-255B strain crossed to the β_1_ or β_2_ RNAi strains were aged for 5 days in the absence or presence of RU486 prior to H_2_O_2_ pretreatment. (**A-D**). The purpose of the experiment was not to completely knockdown the entire pool of 20S proteasome, but only to block the transcription/translation-dependent adaptive increase in proteasome expression following hydrogen peroxide pretreatment. Thus, we used RNAi conditions that blocked increased proteasome expression, without depressing basal proteasome protein levels. Using this approach, we found at least a 50% decrease in mRNA in RNAi strains, and within proteasome western blots and activity, we found blockage of the adaptive increase. After pretreatment, proteolytic capacity of the individual subunits of the 20S proteasome (trypsin-like, caspase/peptidyl glutamyl-peptide hydrolyzing-like activity, and chymotrypsin-like activity) were measured in whole fly lysate. (**A-B**) Proteolytic capacity in β_1_ RNAi flies in the absence (black) “control” or presence (pink in females or blue in males, denoted with “+RU486”) of RU486. (**A**) Females. (**B**) Males. (**C-D**) Proteolytic capacity in β_2_ RNAi flies in the absence (black) “control” or presence (pink in females or blue in males, denoted with “+RU486”) of RU486. (**C**) Females. (**D**) Males. (**E,G**) Females of the β_1_ and β_2_ RNAi strains raised in the absence of RU486 were either not pretreated “control” (black circle) or were pretreated with either 10μM H_2_O_2_ (grey squares) or 100μM H_2_O_2_ (grey circles) for 8 hours, followed by a 16-hour recovery prior to H_2_O_2_ [4.4M] challenge. Females of the β_1_ and β_2_ RNAi strains raised in the presence of RU486 were either not pretreated “+RU486” (pink triangle) or were pretreated with either 10μM H_2_O_2_ (pink diamonds) or 100μM H_2_O_2_ (pink squares) for 8 hours, followed by a 16-hour recovery prior to H_2_O_2_ [4.4M] challenge. (**F,H**) Males of the β_1_ and β_2_ RNAi strains raised in the absence of RU486 were either not pretreated “control” (black circle) or were pretreated with either 10μM H_2_O_2_ (grey circles) or 100μM H_2_O_2_ (grey triangles) for 8 hours, followed by a 16-hour recovery prior to H_2_O_2_ [4.4M] challenge. Males of the β_1_ and β_2_ RNAi strains raised in the presence of RU486 were either not pretreated “+RU486” (green circle) or were pretreated with either 10μM H_2_O_2_ (blue square) or 100μM H_2_O_2_ (blue circle) for 8 hours, followed by a 16-hour recovery prior to H_2_O_2_ [4.4M] challenge. Statistical difference in survival (p < 0.05) was calculated using the Log-Rank test. Statistical summary is located in Supplementary Table S2.

To assess the role of the 20S proteasome in the adaptive response, the ‘Gene-Switch’ system was employed to limit the adaptive increase of the 20S proteasome beta subunit expression [62]. Males of the RNAi strains for the β_1_ and β_2_ subunits were mated to virgin females of the Actin-255B driver strain and adult progeny were raised in the absence or presence of RU486. After continual RU486 exposure, mRNA levels decreased by at least 50% in both sexes (Supplementary Fig. S2A, S2B, S2C, and S2D). In addition, the adaptive increase in the amount of the 20S proteasome was blunted in females upon RU486 exposure (Supplementary Fig. S2E and S2G). Males showed no change in the amount of proteasome expression following H_2_O_2_ pretreatment or RU486 exposure (Supplementary Fig. S2F and S2H). Induction of the proteolytic capacity of the individual subunits was also blocked in females cultured in the presence of RU486 following H_2_O_2_ pretreatment (Fig. 9A and 9C), whereas males showed no change in proteolytic capacity (Fig. 9B and 9D). Similarly, the adaptive response of the 20S proteasome was prevented in females cultured on RU486 and pretreated with H_2_O_2_ (Fig. 9E and 9G). The purpose of these experiments (Fig. 9 and Supplementary Fig. S2) was not to completely knockdown the entire pool of 20S pro-teasome, but only to block the transcription/translation-dependent adaptive increase in proteasome expression following hydrogen peroxide pretreatment. Thus, we used RNAi conditions that blocked increased proteasome expression, without depressing basal proteasome protein levels. Using this approach, we found at least a 50% decrease in mRNA in RNAi strains, and within proteasome western blots and activity, we found blockage of the adaptive increase. Although males showed no change in the adaptive response upon pretreatment following the loss of either the β1 or β2 subunit, limiting the adaptive increase of either subunit decreased survival (Fig. 9F and 9H). Importantly, blocking the adaptive increase in proteasome expression in females also blocked the adaptive increase in survival against a subsequent toxic H_2_O_2_ exposure (Fig. 9E and 9G).

### Decline in 20S proteasome adaptation is accompanied by an accumulation of oxidized proteins

Next, as increased 20S expression and activity was shown to occur in pretreated lysate, it was important to assess whether an inductive increase in the levels of functional 20S proteasome would impact the clearance of oxidized proteins. Indeed, early studies suggested that increased proteasome expression resulted in decreased accumulation of damaged proteins [63]. As well, prior studies in cell culture demonstrated increased protein removal in cells pretreated with an adaptive dose prior to being subjected to a challenge dose of an oxidant [53, 64, 65].

**Figure 10:**
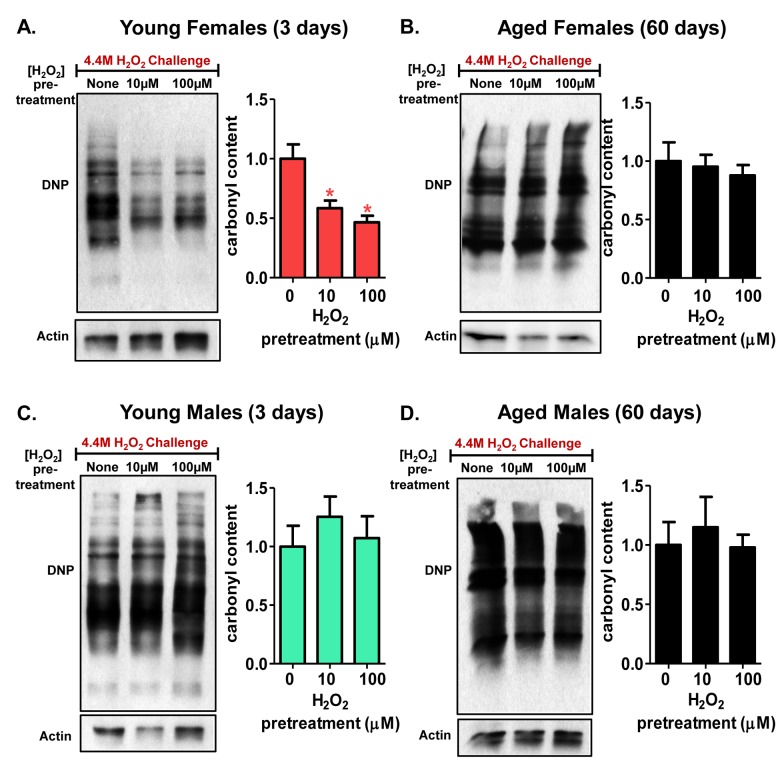
Decline in 20S proteasome induction is accompanied by an accumulation of oxidized proteins Carbonyl content was detected with a DNP antibody in progeny of the Actin-GS-255B strain crossed to w[1118] strain following [0, 10, or 100μM] H_2_O_2_ pre-treatment for 8 hours, followed by a 16-hour recovery to allow for adaption before challenged with H_2_O_2_ [4.4M] for an additional 24 hours. (**A**) Carbonyl content showed significant decrease following H_2_O_2_ pretreatment in 3 day old females. (**B**) Carbonyl content showed no significant change in 60 day old females, irrespective H_2_O_2_ pretreatment. (**C**) Carbonyl content was measured in 3 day old males that were pre-treated with H_2_O_2_. (**D**) 60 day old males showed no change in carbonyl content upon H_2_O_2_ pre-treatment and subsequent recovery. Western blots were performed in triplicate and carbonyl content was normalized to Actin-HRP. Error bars denote standard error of the mean (S.E.M) values. * *P* <0.05, ** *P* <0.01, *** *P* < 0.001, relative to the young control using one-way ANOVA. Statistical significance is shown with asterisks (pink *) in young females.

Having shown the inducibility of both protein expression and activity of the 20S proteasome, following H_2_O_2_ pretreatment of female flies (Fig. 3 and Fig. 4), it was important to determine if the adaptive increase in proteasome expression would limit damage accumulation upon subsequent exposure to a (much higher) toxic amount of H_2_O_2_ [4.4M]. Western blotting was used to examine protein carbonylation levels. Protein carbonylation is an irreversible protein modification, resulting from oxidative damage, that often leads to loss of protein function [66]. Pretreatment with a very low, adaptive dose of H_2_O_2_ [10μM or 100μM] resulted in a much lower accumulation of total protein carbonyls when 3 day old females were subjected to the toxic H_2_O_2_ dose, compared with matched controls that were not pretreated before H_2_O_2_ challenge (Fig. 10A). This was presumably because the increased proteasomal capacity of adapted animals allowed for increased rates of degradation of the oxidized, carbonyl containing, proteins generated during exposure to the toxic dose of H_2_O_2_. However, 60 day old females demonstrated no adaptive decrease in the accumulation of protein carbonyl levels after pre-treatment with toxic levels of H_2_O_2_ (Fig. 10B), following a similar trend in the loss in proteasome induction with age. Similarly, pretreatment with low levels of H_2_O_2_ failed to prevent the significant accumulation of carbonylated proteins in either young or old male flies (Fig. 10C and 10D), both of whom also failed to adapt to H_2_O_2_ in any other way measured in this study. Thus, these results for cumulative protein damage (carbonyl accumulation) reflect the changes (or lack of change) in 20S proteasome expression and activity observed in pretreated flies (Fig. 3A, 3B, and Fig. 4).

### Loss of proteasome subunits or proteasome regulators impacts lifespan

**Figure 11:**
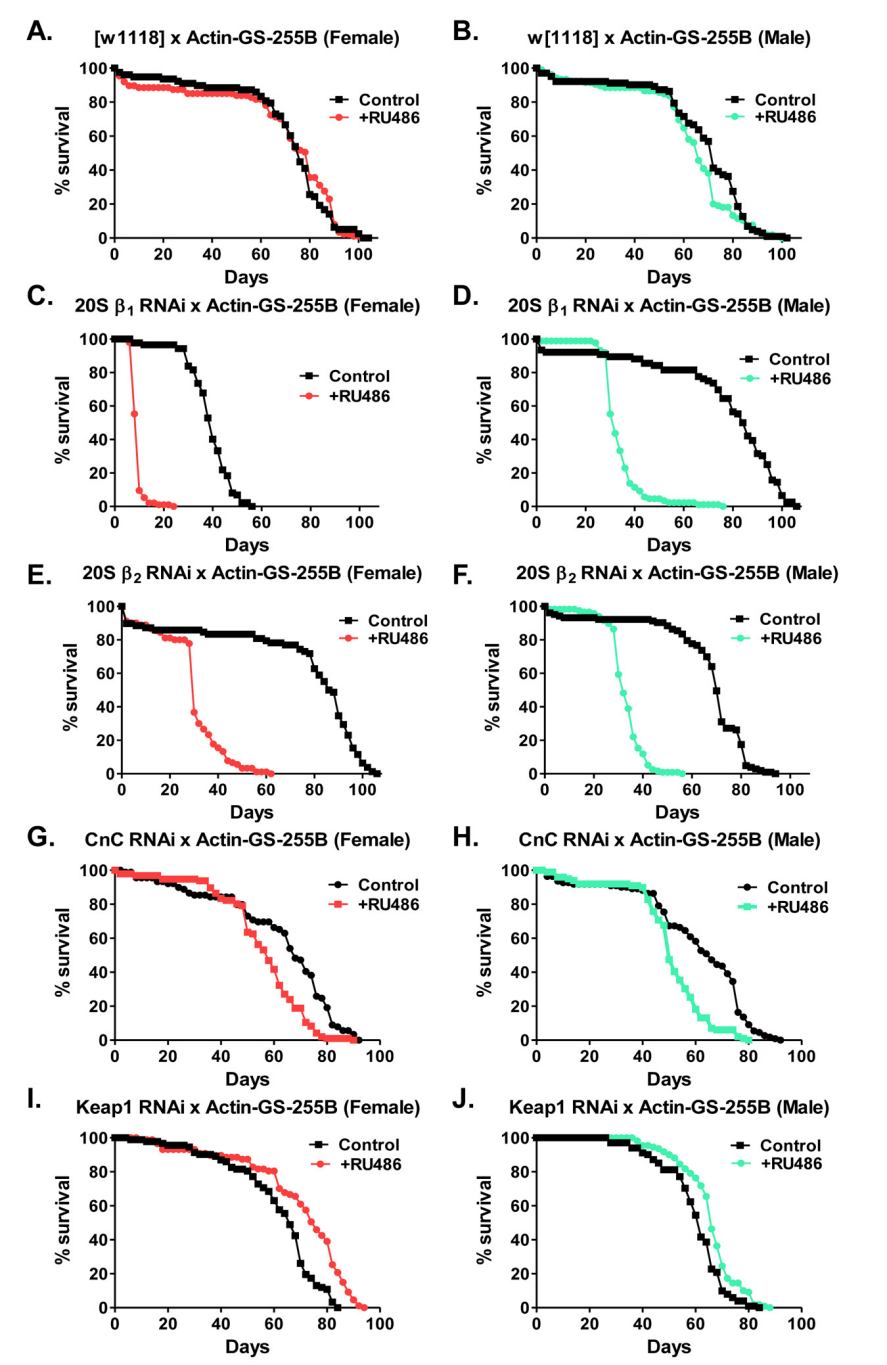
Loss of proteasomal subunits or regulators impacts lifespan (**A,B**) To control for the effect of RU486 on males and females, lifespan of progeny from the Actin-GS-255B strain crossed to w[1118] strain raised in the absence/control (black line) or presence of RU486, pink line for females and blue line for males. (**A**) Females. (**B**) Males. (**C-F**) Effect of removal of proteasome subunits on life span. The Actin-GS-255B strain was crossed to the β_1_ RNAi or β_2_ RNAi strains and the progeny were assayed for life span in the absence/control (black line) or presence (pink line in females and blue line in males) of RU486, as indicated. (**C**) β1 RNAi females. (**D**) β1 RNAi males. (**E**) β2 RNAi females. (**F**) β2 RNAi males. (**G,H**) Effect of removal of the Cap-n-collar (CncC)/Nrf2 orthologue upon lifespan. The Actin-GS-255B strain was crossed to the CncC RNAi strain. Male and female lifespan was measured in the absence/control (black line) or presence (pink line for females and blue line for males) of RU486. (**G**) CncC RNAi female. (H) CncC RNAi male. (I,J) Effect of removal of Keap1 upon lifespan. The Actin-GS-255B strain was crossed to the Keap1 RNAi strain. Males and female lifespan was assessed in the absence/control (black line) and presence (pink line for females and blue line for males) of RU486. (**I**) Keap1 RNAi female. (**J**) Keap1 RNAi male. Statistical difference in survival (p < 0.05) was calculated using the Log-Rank test. Statistical summary is located in Supplementary Table S3.

Previous studies in yeast have shown that loss of the 20S proteasome dramatically decreases lifespan [67]. Additional work has also shown that continual feeding of proteasome inhibitors dramatically reduces lifespan [68]. Moreover, as these results indicate that limiting the induction of the 20S proteasome, blocks adaptation in a sex-specific manner (Fig. 9 and Supplementary Fig. S2), the impact of limiting expression of proteasomal subunits or proteasome regulators upon *Drosophila* lifespan was addressed. The 20S RNAi β1 and β2 strains were utilized to characterize the impact of decreased 20S expression on lifespan. The CncC and Keap1 RNAi strains were also used. Virgin flies were cultured in the presence or absence of RU486 for the entirety of their lifespan [48, 69]. The constitutive feeding of RU486 had no impact on lifespan in control flies (Fig. 11A and 11B). Moreover, the RU486 induced limitation of either β1 or β2 subunit expression (pink in females or blue in males) was detrimental to lifespan in both sexes (Fig. 11C, 11D, 11E, 11F, and Supplementary Table S2). Next, as Nrf2/CncC has been indicated to transcriptionally activate the 20S expression [43, 51], the impact of limiting the expression of CncC upon the lifespan was explored. Upon removal of CncC, both males and females showed a marked decrease in lifespan, albeit not as detrimental as upon direct removal of the 20S beta subunits (Fig. 11G, 11H and Supplementary Table S2). Conversely, upon removal of Keap1, which normally sequesters Nrf2/CncC in the cytosol, males and females showed a slight increase in lifespan (Fig. 11I, 11J and Supplementary Table S2), matching earlier findings [70].

### Stress resistance of aged (60 day old) flies improves upon continual loss of Keap1

**Figure 12:**
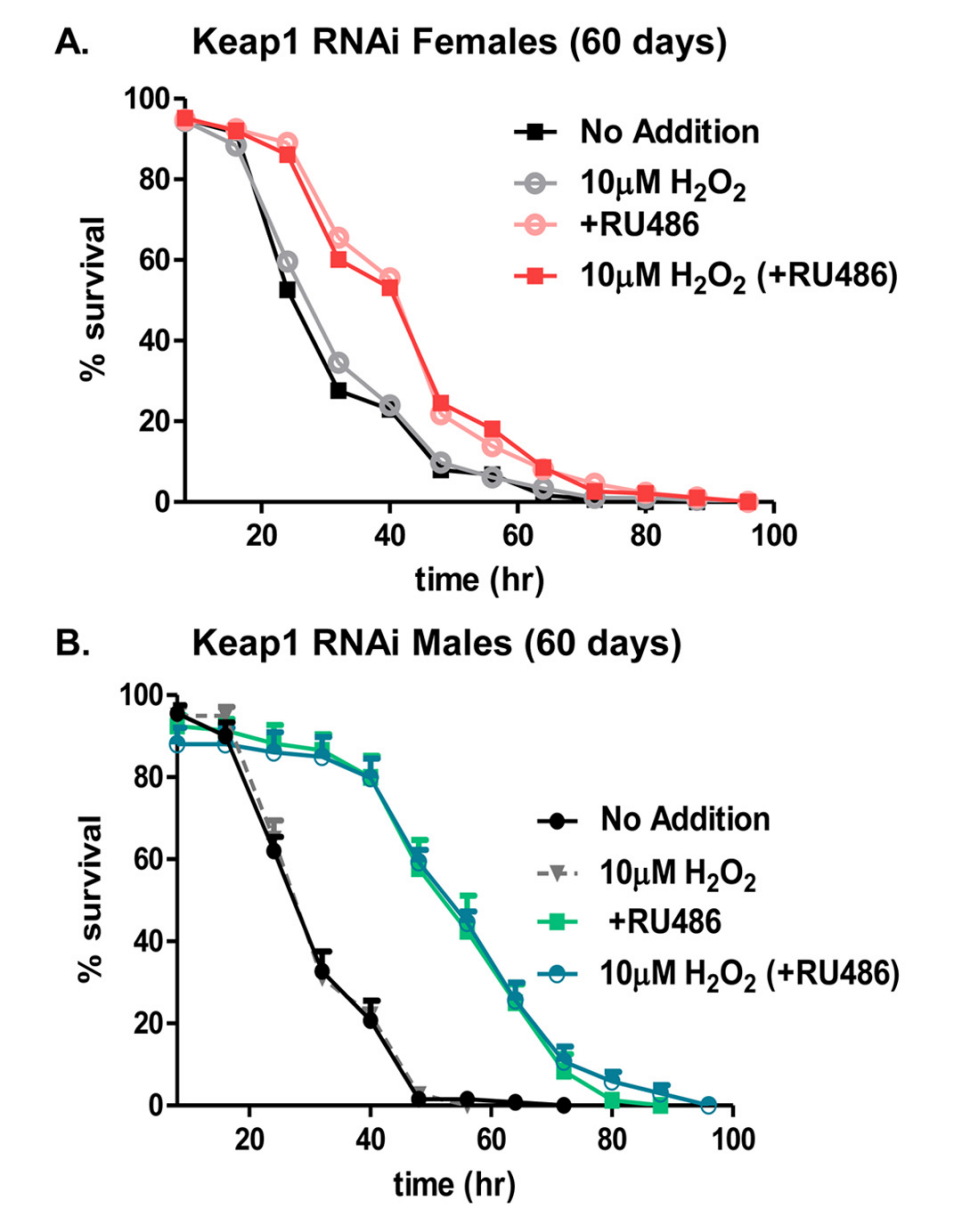
Hydrogen peroxide stress resistance improves with continual knockdown of Keap1 in aged (60 days) flies In panels **A** and **B**, male and female progeny of the Actin-GS-255B strain crossed to Keap1 RNAi strain were collected and aged to 60 days. (**A**) Females raised in the absence of RU486 were either pretreated with 10μM H_2_O_2_ (gray line) “10μM H_2_O_2_” or not pre-treated with H_2_O_2_ (black line) “No Additions”. Females raised in the presence of RU486 were either pretreated with 10μM H_2_O_2_ (red line) “10μM H_2_O_2_ (+RU486)” or not pretreated with H_2_O_2_ (pink line) “+RU486”. (**B**) Males raised in the absence of RU486 were either pretreated with 10μM H_2_O_2_ (gray line) “10μM H_2_O_2_” or not pre-treated with H_2_O_2_ (black line) “No Additions”. Males raised in the presence of RU486 were either pretreated with 10μM H_2_O_2_ (blue line) “10μM H_2_O_2_ (+RU486)” or not pretreated with H_2_O_2_ (green line) “+RU486”. Statistical difference in survival (p < 0.05) was calculated using the Log-Rank test. Statistical summary is located in Supplementary Table S4.

Adaptive homeostasis [8] is necessary for dynamic regulation of protein quality control. As the 20S proteasome is an integral mechanism for protein turnover, loss of its inducibility, in response to mild oxidative stress, is an indicator of protein dysregulation [53]. The loss of 20S proteasome induction with age is potentially attributed to changes in Nrf2/CncC transcriptional regulation, as this transcription factor determines 20S proteasome expression upon oxidative insult [43, 51, 68, 70]. To begin to address this disparity between chronic elevation of the 20S proteasome and its dynamic response to stress, an earlier study explored the impact of chronic over-expression of the *C. elegans* Nrf2/CncC homolog, Skn-1, throughout the lifespan, and found improved stress tolerance, yet inability to activate the proteasome adaptive response with age [53]. In turn, indicating a dichotomy between chronic basal expression and transient induction of the proteasome. Additionally, RNAi studies against Keap1, the cytosolic inhibitor of CncC, resulted in an elevation of proteasomal subunits [57]. Therefore, the continual removal of Keap1, throughout the lifespan, was employed to determine whether chronic knockdown would be beneficial in mediating an adaptive response in aged flies. Male and female flies of the Keap1 RNAi strain were aged, continually, in the absence (blue) or presence (pink) of RU486. After 60 days, flies were used as controls/no pretreatment, or were pretreated with 10μM H_2_O_2_, prior to being challenged with 2M (females) or 1M (males) H_2_O_2_. Unfortunately, adaptation (increased survival) was not restored upon continual knock-down of Keap1 following H_2_O_2_ pretreatment, compared to flies with normal Keap1 levels (Fig. 12A and 12B). However, comparison between flies fed only the challenge dose, showed overall stress resistance was improved upon constant Keap1 removal in females and males (Fig. 12A and 12B). Nor was this improved stress resistance due to RU486, alone, as control males and females, aged continually in the absence or presence of RU486, showed no difference in stress resistance (Supplementary Fig. S3). Together showing that although adaptation was not restored, stress resistance was improved by lifelong Keap1 knockdown. Therefore, further solidifying the disconnect between the adaptive and basal changes of the stress response.

## DISCUSSION

Physiological Adaptive Homeostasis has been defined as, “The transient expansion or contraction of the homeostatic range in response to exposure to sub-toxic, non-damaging, signaling molecules or events, or the removal or cessation of such molecules or events” [8]. Transiently expanding the homeostatic range involves the temporary activation or induction of cytoprotective genes and pathways that enable an organism to withstand exposures to toxic agents or situations that would normally be damaging or even lethal. Such protective adaptive responses are by nature short-lived, and disappear within hours. Studies in mammalian cell culture have demonstrated the adaptive increase of the 20S proteasome, following exposure to signaling events (including H_2_O_2_, heat shock, and nutrient starvation) [51, 71]. More importantly, adaptation is found to be conserved in higher organisms, including *C. elegans* and* D. melanogaster*, where exposure to short-term H_2_O_2_, mild heat shock, 100% oxygen or irradiation, have been found to increase survival [43, 44, 53, 72-74] against serious stresses. With advancing age, however, the dynamic range of adaptation, or ‘adaptive homeostasis’ [8], shrinks. In consequence, aging has been characterized as the chronic activation of stress responsive pathways [11]. Here, both the age-associated and sex-specific loss of the adaptive capacity of the 20S proteasome, as well as stress resistance and longevity, was explored in *D. melanogaster* flies.

The 20S proteasome is the primary mechanism for immediate removal of oxidized proteins following stress [5, 75, 76]. Therefore, it is important to assess the *adaptive* homeostatic capacity of the 20S proteasome. Three day old H_2_O_2_ pretreated females were capable of increased 20S proteasome expression, increased proteolytic capacity, and survival, which was lost with age. A finding that is consistent in the nematode worm [53]. Moreover, it was found that adaptive increases in proteolytic capacity depends on the 20S proteasome, as blocking induction in whole animals, or inhibition of activity in pretreated fly lysates, eliminated increased proteolysis

A key measure of age-related changes in proteolytic function is protein degradation. While, the general consensus is in accord with the amount and activity of the catalytic core declining with age [77], it is not universal [20, 61], and is highly tissue-dependent [57, 78]. Moreover, earlier studies indicate an age-associated increase in the basal amounts of the 20S proteasome in rat muscle [79, 80], *C. elegans* [53], and as presented here, in *D. melanogaster*. These findings substantiate prior studies that suggested a similar trend in the dysregulation of proteasome expression and activity with age. For example, post-mitotic aging in human lung fibroblasts demonstrated loss of chymotrypsin-like proteasomal activity [77]. In addition, tissue-specific declines in proteolysis have been reported in a plethora of tissues, including the brain [81], liver [82], heart [83], and retina [59], albeit, findings differ and vary between the catalytic subunits. This is potentially attributed to the increased accumulation of oxidized and cross-linked aggregates of proteins with age, which have been shown to bind to proteasome but which cannot be degraded; thus they act as very effective and irreversible proteasome inhibitors [84].

Furthermore, highly oxidized proteins are no longer ideal proteasome substrates, as heavily aggregated proteins have been shown to inhibit the 20S catalytic core [55, 75, 85]. Supported by recent work to elucidate the ‘insolubolome’, or proteins that become insoluble with age, subunits of the 20S proteasome were found to actually accumulate with these protein aggregates [13]. Thus to cope with increased protein damage, organisms may upregulate 20S proteasome expression but, proteasomes may become irreversibly bound to such aggregates, and accumulate, thus adding to overall protein aggregation, rather than removal. Proteasomes bound in such aggregates of cross-linked, oxidized proteins would add to the total amount of proteasome expression, but not to proteasomal activity. Evidence for this hypothesis has been explored in the brains of Alzheimer's disease (AD) patients, as protein aggregates, in the form of plaques and tangles, is arguably a contributing factor in disease progression [86]. Additionally, amyloid-beta has been suggested to inhibit proteasome activity [87]. Indeed various studies assessing changes in proteasome, indicate it is not a decline in proteasome expression, but rather a loss in proteolytic activity that may contribute to disease manifestation [88, 89].

In this study, the age-related dichotomy between the *amount* versus the *proteolytic capacity* of the 20S core proteasome was found to be consistent in aged fruit-flies. Here, aged females showed an age-associated basal increase in 20S expression. Yet, proteolytic capacity of the three catalytic subunits (caspase-like, trypsin-like, and chymotrypsin-like) showed a significant decline in whole-body lysates from 60-day old flies. A finding mirrored upon the addition of physiologically-relevant substrates, specifically oxidized proteins [75]. Using oxidized hemoglobin as a substrate, an increase in proteolytic capacity was observed only in lysates from young pretreated females, which was lost in aged pretreated females. Males showed no change in proteolytic capacity, regardless of age or pretreatment. Indeed, the importance of the 20S proteasome catalytic core was highlighted by the dramatic shortening of lifespan that was evident, in both sexes, upon the removal of the catalytically active beta subunits, indicative of the crucial role of the 20S proteasome in protein turnover. Together, these findings suggest a maximal threshold that is reached in proteasome expression as a possible age-dependent compensatory mechanism. Indeed, the narrowing of the homeostatic range is a conserved feature of aging, as chronic activation of the stress response is evident in both aged mammalian tissues and model organisms [11, 53, 90].

As suggested by earlier studies, the activity of the three catalytic subunits of the 20S core proteasome are subject to tissue-dependent variation [57, 58, 60, 78, 91]. However, to our knowledge, tissue-specific changes in the adaptive response, with age, have not been explored. Here, young females, pretreated with H_2_O_2_, showed increased proteolytic activity in all three beta-subunits within the abdomen and head. Increased proteasome activity mirrored the inductive increase in the tissue-specific expression of the 20S proteasome core. No change in thoracic tissue was evident, for reasons that are not yet clear. It might be interesting to try direct thoracic injection of H_2_O_2_ to determine if this tissue is capable of adapting [92]. Notably, in contrast with females, males showed no increase in proteolytic activity following H_2_O_2_ pretreatment, irrespective of which tissue was examined.

Although induction of proteasome expression and activity was lost in aged females, the tissue specific differences in the basal proteasomal catalytic activities was still very interesting. Female head, abdomen, and thorax all displayed an increase in basal caspase-like activity. Conversely, basal chymotrypsin-like activity showed a significant decrease with age in the abdomen, head, and thorax, whereas trypsin-like activity declined only in the abdomen. Interestingly, males exhibited a similar pattern in individual basal proteolytic activities, with a significant increase in the basal caspase-like activity, coupled with a significant decrease in basal chymotrypsin-like activity in all three tissues.

The significant declines in basal chymotrypsin-like activity in all three tissues, for both females and males, is particularly relevant as studies using site-specific mutagenesis and site-specific inhibitors found the chymotrypsin-like activity to be the rate-limiting step for protein degradation [93, 94]. This finding is given further significance by studies of substrate interaction, which demonstrate that substrates which bind to proteasome's chymotrypsin-like site cause the activa-tion of the caspase-like site, but not vice versa [95]. More importantly site-directed mutagenic inactivation of the proteasomal β5 subunit, responsible for chymotrypsin-like activity, resulted in cells that became hypersensitive to oxidative stress [96]. Additionally, certain proteolytic activities may be better protected, as shown following overexpression of the heat shock 90 chaperone, which ensured retention (and even improvement) of the trypsin- and caspase-like activity [97], suggesting a synergistic protective pathway may arise with age. Moreover, the dichotomy within the individual catalytic subunits of the proteasome is evident in aging tissues, including the brain [81], liver [82], heart [83], and retina [59], albeit, findings differ and vary between the catalytic subunits. Indeed, a similar outcome is presented here, as aged flies show increased sensitivity to hydrogen peroxide, paralleled by a dramatic decrease in chymotrypsin-like activity. Thus the loss of chymotrypsin-like activity may quite possibly have the greatest detrimental impact upon protein turnover, and ultimately cell survival.

Another highly relevant finding is the sex-specific difference in proteasome responses. Initial D. melanogaster studies showed age-related declines in basal proteolytic activity, but did little to address sex-dependent differences as they used mixed populations of flies [20]. A later study found female D. melano-gaster to have higher basal proteolytic activity of both the 26S and 20S proteasomes compared to males [57]. Moreover, the basal disparity in proteasome activity has been found to extend to rats [97] and humans [98], as livers from aged females appear to exhibit higher basal chymotrypsin-like activity than do livers from aged males. However, as the 20S proteasome is the primary defense for immediate removal of oxidized proteins, it is important to assess the sex-specific differences in its *adaptive* homeostatic capacity. Here, it was demonstrated that although young males and females displayed similar stress resistance upon increasing amounts of H_2_O_2_, males showed no difference in survival following H_2_O_2_ pretreatment, reiterating an earlier finding from our lab [43, 47]. Nor is this difference dependent upon pretreatment dosage, as increasing amounts of hydrogen peroxide have been previously shown only to be detrimental to males [43]. In addition, only young females exhibited induction of proteasome synthesis with H_2_O_2_ pretreatment, and decreased accumulation of oxidized proteins, whereas H_2_O_2_ pretreatment of males had no measurable effect on any of these parameters. Nor are sex-differences diminished with age, as aged males were less stress-resistant than were aged females.

Understanding sex-dependent differences in adaptive homeostasis, including stress survival, proteasome inducibility and activity, may be crucial in understanding the underlying cause(s) for sex-dependent differences in healthspan and lifespan. Females typically outlive males, as evident in fruit-flies [35, 98], rodents [99], and humans [100]. Moreover, the higher basal activities of nuclear-encoded antioxidant genes in females, may account for the four-fold higher damage accumulation present in males [101, 102]. Also, studies (including this one) suggest that females have both a higher basal proteolytic capacity and a more inducible proteasome compared to males [57, 103]. Nor are sex-differences diminished with age, as aged males were less stress-resistant than were aged females. Thus it is plausible females are more efficient at modulating stress responses, including proteasome expression and activity.

Prior work has shown that knock-down of the Nrf2 orthologues, CncC (fly) or Skn-1 (worm), abrogates the adaptive response to H_2_O_2_, including both the increase in H_2_O_2_ resistance and the increase in proteasome expression [43]. In attempt to restore the adaptive response in aged females, chronic knockdown of Keap1 was tested. Simplistically, Keap1 binds CncC in the cytoplasm, preventing its nuclear translocation and subsequent activation of target genes (including the proteasome). Though it proved unsuccessful to restore adaptation, it did significantly increase basal resistance to H_2_O_2_ toxicity. A similar outcome arose upon chronic overexpression of Nrf2/Skn-1 in the nematode worm [53]. Together, findings in both worms and flies, indicate that simply overexpressing Nrf2 or suppressing Keap1 in aged animals is insufficient to restore the highly dynamic processes involved in adaptive homeostasis. Nevertheless, both strategies did significantly increase baseline stress resistance, which may be an important determinant of healthspan and/or lifespan.

Overall, these findings highlight the importance of understanding the age-related and sex-specific changes in the adaptive stress responses of the 20S proteasome. Further work will be needed to fully uncover the underlying mechanisms for the loss of adaptive homeostasis in aged animals, and to (potentially) find means for its restoration.

## METHODS

### Drosophila strains and culture

Flies were cultured on standard agar/dextrose/corn meal/yeast media at 25°C as described previously [69]. Flies expressing RNAi against two proteasome subunits were obtained from the Vienna *Drosophila* RNAi center (VDRC, Vienna, Austria), *w*[1118]*; P[GD13913]v35923* (abbreviated B_1_ RNAi), *w*[1118]*; P[GD10938]v24749* (abbreviated B_2_ RNAi). Strains expressing RNAi against the cap ‘n’ collar transcription factor (an orthologue of the mammalian Nrf2) and dkeap-1 (an orthologue of the mammalian Keap1) were kindly donated by Dr. Dirk Bohman [70, 104]. Males from these lines (or *w*[1118] as a control) were crossed to virgin females of the Actin-‘Geneswitch’-255B (Actin-GS-255B) driver strain [62]. Virgin progeny were collected over 48 hours following eclosion. Adult flies were maintained on media adjusted to final concentration of 160μg/mL mifepristone (RU486, no. M8046, Sigma-Aldrich) or ethanol, and transferred to fresh media every other day.

### Preparation of Drosophila

Twenty flies were collected per treatment group and re-suspended in 200μL proteolysis buffer (50mM Tris/HCl, 20mM KCl, 5mM MgAc, 1mM DTT, pH 7.5) and homogenized using an electric pestle. Further lysis was performed by three ‘freeze-thaw’ cycles, which consisted of 5min incubation on dry ice, followed by 5min incubation in water, and vortexed. To remove cuticle fragments, samples were centrifuged for 10,000g for 10min at 4°C. Protein concentration was measured using the Bicinchoninic acid assay (BCA) reducing agent compatible kit (no. 23252, Thermo-Scientific).

### Western blots

Protein samples were run on a 4-15% gradient SDS-PAGE gel (no. 4568084, Bio-Rad) and transferred to a PVDF membrane (no. 1620177XTU, Bio-Rad). Mouse monoclonal antibody specific for detection of the α subunit of the 20S core of *D. melanogaster* origin (1:100 dilution, no. sc-65755, Santa Cruz Biotechnology). The goat polyclonal anti-Actin-HRP antibody, conjugated to horseradish peroxidase (1:1000 dilution, no. sc-1616, Santa Cruz Biotechnology) was used for protein loading control.

### Fluoropeptide proteolytic activity assays

5μg of lysate from treatment groups were transferred, in triplicate, to 96-well plates, and 2μM of various proteasome specific-subunit substrates were used: Caspase-like/β_1_ activity, Z-LLE-AMC (no. 539141, Calbiochem), Trypsin-like/β_2_ activity, Z-ARR-AMC (no. 539149, Calbiochem) Chymotrypsin-like/β_5_ activity, Suc-LLVY-AMC (no. 539142, Calbiochem). Plates were incubated at 37°C, and fluorescence readings were recorded every 10 minutes for 4h using an excitation wavelength of 355nm and an emission wavelength of 444nm. Fluorescence units were converted to moles of free 7-amino-4-methylcoumarin (AMC), using an AMC standard curve of known amounts (no. 164545, Merck), with background subtracted. To measure proteolytic inhibition, 20μM of the proteasome inhibitors, lactacystin (no. 80052-806, VWR) or epoxomicin (no. E3652, Sigma-Aldrich), were added directly to lysate, and incubated on plate shaker for 30min at 300rpm, after which, substrate was added.

### Preparation of [^3^H]-labeled substrates

Tritium-tagged oxidized-hemoglobin ([^3^H]-OxHb) was generated as previously described [6]. Briefly, 5mg of hemoglobin was dissolved in 0.1M Hepes buffer with the addition of 6.6uCi [H3]Formaldehyde and 20mM sodium cyanoborohydride [105]. Mixture was incubated at room temperature on an end-over-end shaker for 1 hr. Hydrogen peroxide (H_2_O_2_) was added at a final concentration of 5mM, and mixture was rocked for an additional hour. Mixture was dialyzed through a 10,000 MWCO filter (Millipore) at 15,000g for 30min, eluent was discarded, and slurry re-suspended in Hepes buffer. This was repeated for an additional 7 washes to remove unbound [H3]Formaldehyde. Protein content was quantified with BCA assay kit (Thermo-Scientific).

### [^3^H]-labeled substrates proteolytic activity assay

5μg of oxidized [^3^H] hemoglobin (([^3^H]-OxHb) was added to 15μg of cell lysate. Samples were incubated on a plate shaker at 300rpm for 2 hours at 37°C. To precipitate any remaining intact protein, 20% trichloroacetic acid and 2% BSA was added. Samples were centrifuged at 13,000rpm for 10min and supernatant was collected and added to 10mL of scintillation fluid. The release of acid-soluble counts were read on a scintillation counter. Background was subtracted and the amount of liberated radiolabel was reported.

### RNA isolation and quantitative RT-PCR

RNA isolation was performed following manufacturer's instructions with slight modification. Flies were homogenized in 500μL TRIzol (no.15596-026, Life Technologies) before an additional 500μL TRIzol were added and incubated at room temperature for 5min. Samples were centrifuged at 12,000g for 10min at 4°C to remove cuticle. 200μL of chloroform was added to the supernatant before samples were shaken for 15 seconds, and incubated at room temperature for 5min. Samples were centrifuged at 12,000g for 15min at 4°C. To the aqueous phase, 500μL of ice-cold 100% isopropanol was added, and samples were incubated at room temperature for 10min. To pellet the RNA, samples were centrifuged at 12,000g for 10min at 4°C and supernatant was decanted. To wash the RNA pellet, 1mL of 70% ice-cold ethanol was added, briefly vortexed before being centrifuged at 7500g for 5min at 4°C. The RNA pellet was dried before re-suspended in DEPC-treated water. RNA concentration was measured using a Nanodrop spectrophotometer (Thermo-Scientific).

For cDNA generation, RNA was reverse transcribed using TaqMan® Reverse Transcription Reagents (no. N8080234, Life Technologies). Quantitative PCR was carried out using iTaq SYBR Green (no. 1725120, Bio-Rad). Amplification of the Beta1 subunit was conducted using the following primer sequence (Forward: 5′ CAGTCATTTCGTGTTCGTGC Reverse: 5′ TCGAACTCCACTGCCATAATG). Amplification of the Beta2 subunit was conducted using the following primer sequence (Forward: 5′AGGTGGTGTTATTCTGGGC Reverse: 5′ TCCGTAGTCATCTCAGTGTCC). Primers for Rp49 were used as an internal control (Forward: 5′CGGATCGATATGCTAAGCTGT Reverse: 5′ GCGCTTGTTCGATCCGTA). Primers were designed using the NCBI Primer-Blast software [106].

### Carbonyl content

The protein oxidation detection kit, Oxyblot (no. S7150, Millipore) was utilized to perform immunoblot detection of oxidatively modified proteins. 5μg of protein from young flies (3 days) and aged flies (60 days) was prepared in the same manner as samples for western blot analysis. Carbonyl groups in samples were derivatized to 2,4-dinitrophenylhydrazone (DNP-hydrazone) by reaction with 2,4-dinitrophenylhydrazine (DNPH). Samples were run on a 10% SDS-PAGE gel and transferred to a PVDF membrane for western blot analysis as described previously. Blots were incubated with goat polyclonal anti-Actin-HRP antibody, conjugated to horseradish peroxidase (1:1000 dilution, no. sc-1616, Santa Cruz Biotechnology) to assess protein loading, and detection of carbonylated proteins was measured with the mouse monoclonal anti-DNP antibody (no.MAB2223, Millipore).

### Drosophila hydrogen peroxide challenge assays

Flies were aged for 3 days (young) or 60 days (old) before being transferred onto Kimwipes containing 5% sucrose, 24 hours prior to H_2_O_2_ exposure. Flies were subsequently transferred to vials containing various toxic doses of H_2_O_2_ [0M-8M] and survival was measured. Flies were scored as dead once they became completely immobile [43, 44].

### Drosophila hydrogen peroxide pretreatment

24 hours prior to H_2_O_2_ exposure, flies were transferred to vials containing 5% sucrose on a Kimwipe. For pretreatment (to initiate adaptation), flies were placed for 8 hours in vials without hydrogen peroxide, or in vials that contained 10μM or 100μM H_2_O_2_. Flies were then transferred to vials containing 5% sucrose for 16 hours to allow for an adaptative response to occur. Afterwards, flies were either immediately collected to assess proteasome expression, or were placed into vials containing a toxic dose of H_2_O_2_ and survival was measured to assess adaptation.

### Lifespan assays

Lifespan assays were performed as previously described [107]. Age-synchronized flies of virgin males and females were collected from culture bottles over a 48 hour period following eclosion. 20 females and 25 males were housed per vial. Flies were transferred to fresh media every other day and deaths were recorded. The mean, median, percent change in the mean and median, and the log-rank p value were calculated using the R statistical software [108]. The values are located in the supplementary lifespan tables.

## SUPPLEMENTARY MATERIAL FIGURES AND TABLES



## Abbreviations

*D. melanogaster*: *Drosophila melanogaster*
*C. elegans*: *Caenorhabditis elegans*
H_2_O_2_: hydrogen peroxide
Nrf2: the transcriptional regulator, nuclear factor ethyroid 2-related factor 2
Cnc-C: D. melanogaster cap ‘n’ collar transcription factor (ortholog of mammalian Nrf2)
Skn-1: C. elegans skinhead-1 transcription factor (ortholog of mammalian Nrf2)
AMC: 7-amino-4-methylcoumarin
