# Supplementary material for: The age- and sex-specific decline of the 20s proteasome and the Nrf2/CncC signal transduction pathway in adaption and resistance to oxidative stress in Drosophila melanogaster
Source: Aging (Albany NY). 2017 Apr 3;9(4):1153–78. doi: 10.18632/aging.101218 (PMC5425120; doi:10.18632/aging.101218)
Supplement: Supplementary file 1 [file aging-09-1153-s001.pdf]

SUPPLEMENTARY MATERIAL

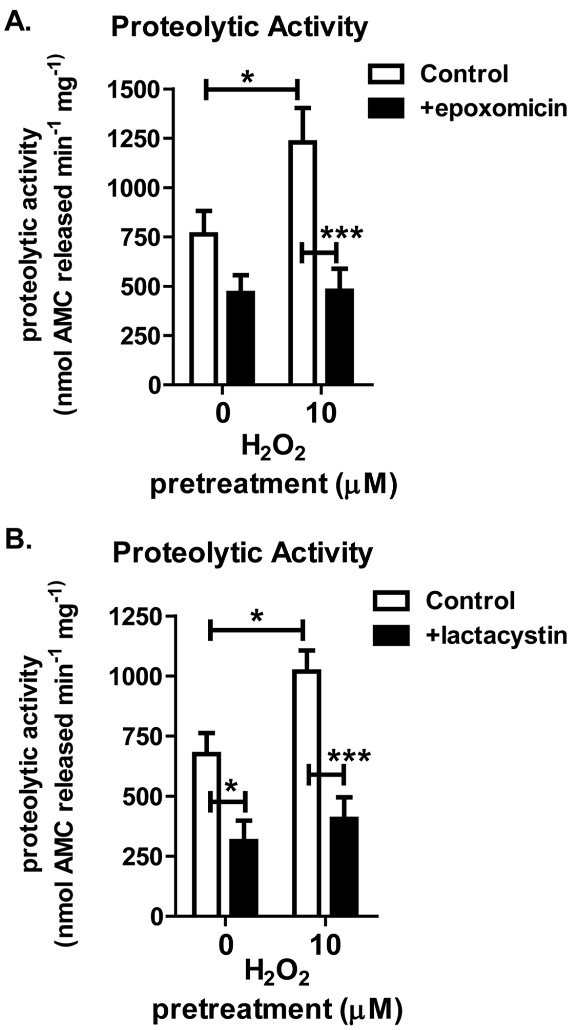

Supplementary Figure S1. Inhibition of the 20S proteasome proteolytic activity in young (3 day old) female flies. To test whether the hydrogen peroxide induced proteolytic activity in young female flies is proteasome dependent, three day old female progeny of the Actin-GS-255B strain crossed to the w[1118] strain were either pretreated with 10µM H2O2or not pretreated with H2O2. Whole flies were homogenized and lysed in proteolysis buffer and sample lysates were then incubated for 30 minutes either alone (white) "control", or with proteasome inhibitors (black). (**A**) epoxomicinor (**B**) lactacystin, prior to the addition of the Suc-LLVY-AMC proteasome fluoropeptidesubstrate. Error bars denote standard error of the mean (S.E.M) values. * P <0.05, and *** P < 0.001, relative to control not treated with inhibitor, using one-way ANOVA.

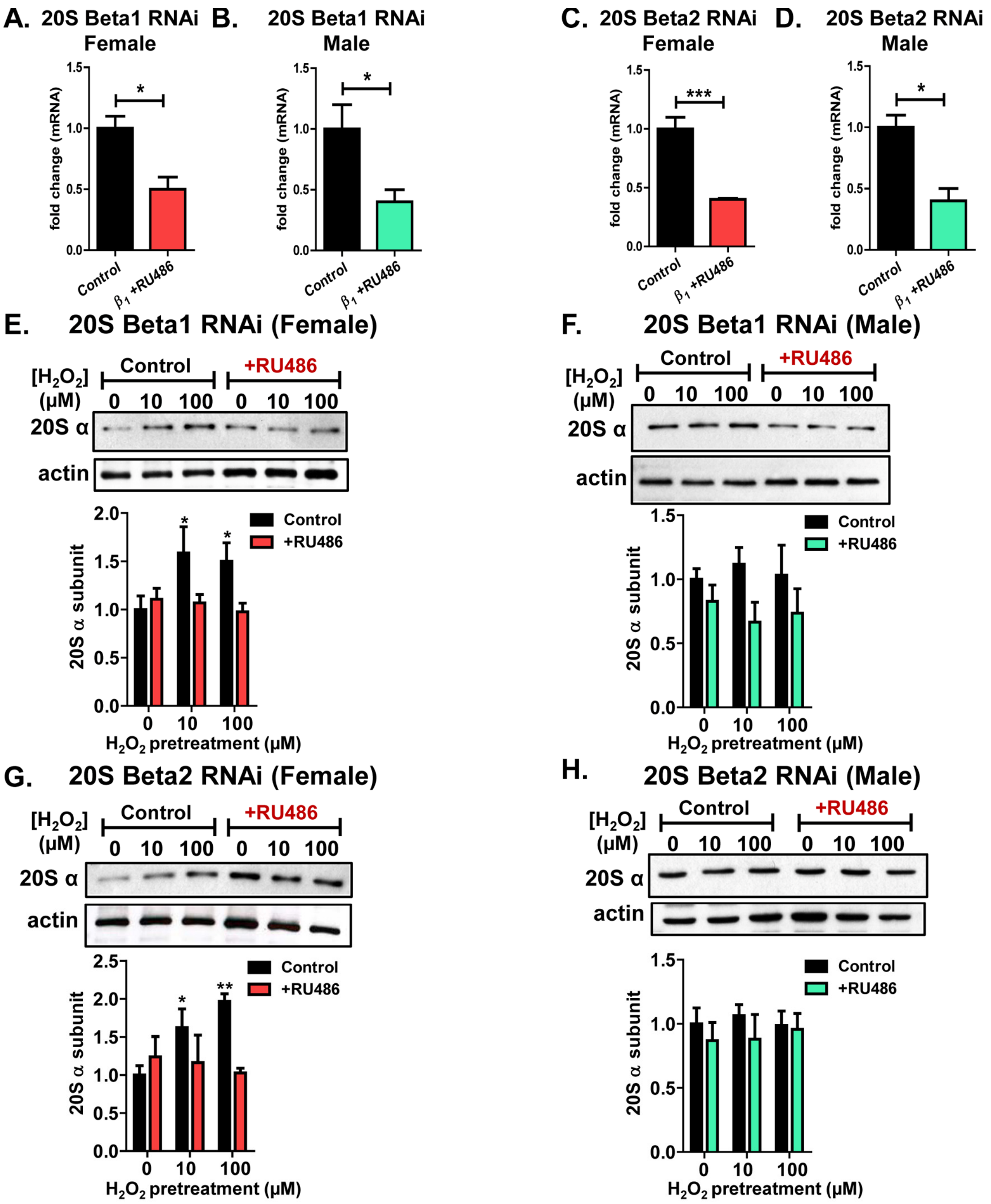

Supplementary Figure S2. Loss of the 20S proteasome abrogates the adaptive response. (**A**-**H**) Progeny of the Actin-GS-255B strain crossed to the β1 or β2 RNAi strains were incubated for 5 days in the absence or presence of RU486, to block the transcription/translation-dependent adaptive increase in proteasome expression following H2O2pretreatment. RNAi conditions blocked increased proteasome expression, without depressing basal protein levels. At least 50% decrease in mRNA in RNAi strains, and within proteasome western blots and activity, blockage of the adaptive increase. (**A**,**C**) In the presence of RU486 (pink), females show 50% decrease in mRNA for both beta subunits. (**B**,**D**) Similarly, in the presence of RU486 (blue), males show at least 50% reduction in the amount of mRNA. (**E**-**H**) Following incubation, in the presence or absence of RU486, flies were either pretreated with adaptive doses of H2O2[10μM or 100μM] or used as controls, before whole flies were collected for analysis of protein expression. (**E**,**G**) In the absence of RU486 (black), pretreated females show a robust increase in the 20Sα expression, which was blocked in RU486-fed females (pink). (**F**,**H**) Irrespective of the absence or presence of RU486, males showed no change in 20Sα expression. Western blots were performed in triplicate, normalized to Actin-HRP. Error bars denote standard error of the mean (S.E.M) values. * P <0.05, ** P <0.01, *** P < 0.001, relative to the control using one-way ANOVA.

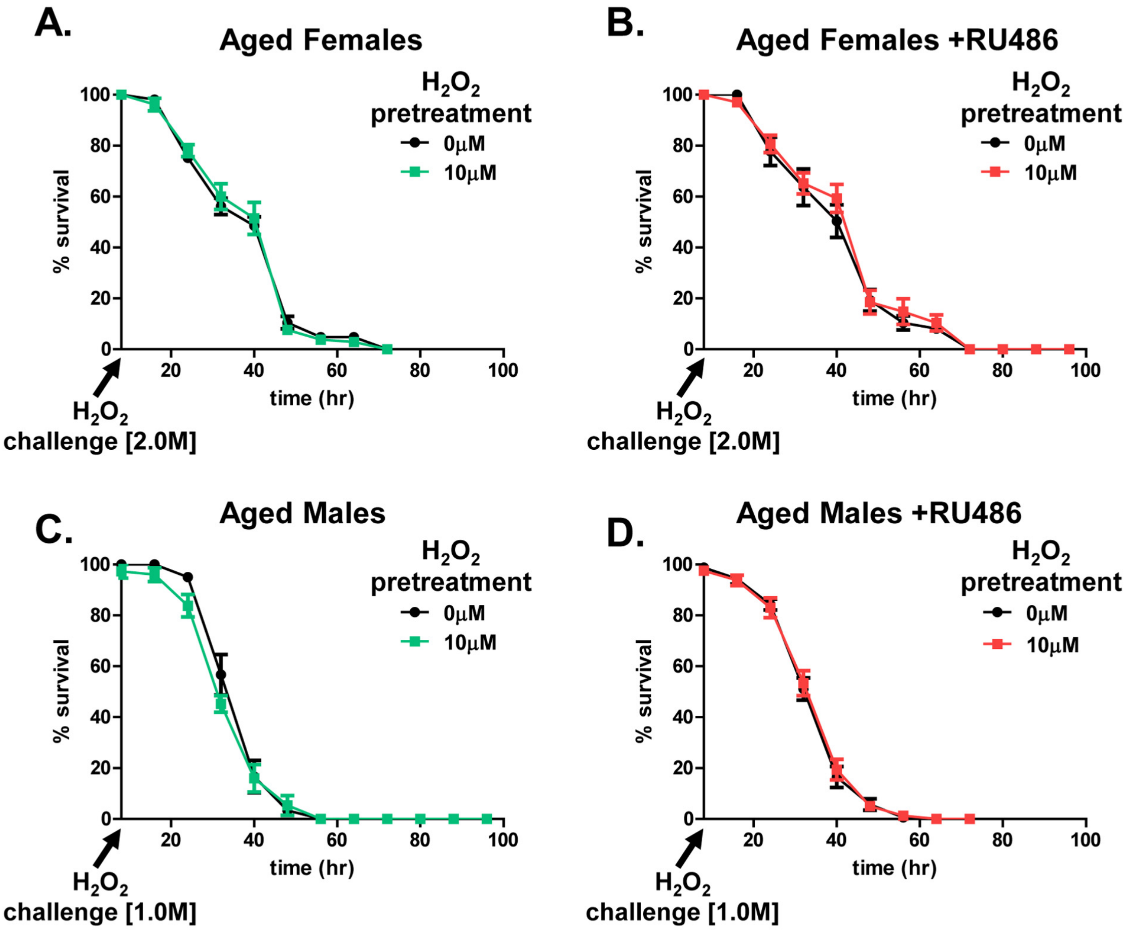

Supplementary Figure S3. RU486 by itself does not affect H2O2 adaptation or resistance in aged (60 day old) male or female flies. (**A**-**D**) Female and male progeny of the Actin-GS-255B strain were crossed to the w[1118] strain aged in the absence or presence of RU486, continually, for 60 days and then used as controls, or given a 10µM H2O2pretreatment, prior to exposure to a 2.0M H2O2challenge (females) or a 1.0M H2O2challenge. (**A**) 60 day old females raised in the absence of RU486. (**B**) 60 day old females raised in the presence of RU486. (**C**) 60 day old males raised in the absence of RU486. (**D**) 60 day old males raised in the presence of RU486. Blue line indicates males and females, raised in the absence of RU486, pretreated with 10µM H2O2. Pink line indicates males and females, raised in the present of RU486, pretreated with 10µM H2O2. Statistical summary is located in Supplementary Table S5.

Supplementary Table S1. Hydrogen peroxide adaptation in 3 day and 60 day old males and females statistical summary. Two cohorts of 3 day old and 60 day old females and males either not pretreated with H2O2or pretreated [10µM] H2O2. Crosses are in order males x virgin females.

Female (Cohort 1)

Genotype	Age (day)	H_2O_2	N	Mean (SD)	Median	90%	Δ Mean %	Δ Median %	(p)
w[1118] x Actin-GS-255B	3	0µM	69	82 (4)	84	104			
w[1118] x Actin-GS-255B	3	10µM	81	94 (4)	95	108	9.42	7.69	0.0009
w[1118] x Actin-GS-255B	60	0µM	97	49 (16)	48	73			
w[1118] x Actin-GS-255B	60	10µM	106	49 (16)	48	73	-0.778	0	0.7997

Female (Cohort 2)

Genotype	Age (day)	H_2O_2	N	Mean (SD)	Median	90%	Δ Mean %	Δ Median %	(p)
w[1118] x Actin-GS-255B	3	0µM	58	53 (16)	55	80			
w[1118] x Actin-GS-255B	3	10µM	69	66 (18)	67	88	15.6	20.0	3.82E-12
w[1118] x Actin-GS-255B	60	0µM	107	48 (16)	48	70			
w[1118] x Actin-GS-255B	60	10µM	90	51 (15)	52	73	3.42	4.76	0.3131

Male (Cohort 1)

Genotype	Age (day)	H_2O_2	N	Mean (SD)	Median	90%	Δ Mean %	Δ Median %	(p)
w[1118] x Actin-GS-255B	3	0µM	73	58 (13)	60	71			
w[1118] x Actin-GS-255B	3	10µM	79	58 (13)	60	71	0.584	0.000	0.7838
w[1118] x Actin-GS-255B	60	0µM	88	33 (14)	34	40			
w[1118] x Actin-GS-255B	60	10µM	93	33 (14)	34	40	-0.406	0	0.9166

Male (Cohort 2)

Genotype	Age (day)	H_2O_2	N	Mean (SD)	Median	90%	Δ Mean %	Δ Median %	(p)
w[1118] x Actin-GS-255B	3	0µM	57	57 (15)	61	76			
w[1118] x Actin-GS-255B	3	10µM	69	56 (15)	60	74	-0.562	0	0.7609
w[1118] x Actin-GS-255B	60	0µM	79	29.9 (16)	28	37			
w[1118] x Actin-GS-255B	60	10µM	80	29.5 (16)	28	37	-0.778	0	0.7997

Supplementary Table S2. Hydrogen peroxide adaptation in Beta1 and Beta2 RNAi strains. Two cohorts of females and males propagated in the absence or presence of RU486. Flies were either not pretreated with H2O2or pretreated [10µM or 100µM] H2O2. Crosses are in order males x virgin females.

Female (Cohort 1)

Genotype	RU486	H_2O_2	N	Mean (SD)	Median	90%	Δ Mean %	Δ Median %	(p)
Beta1 RNAi x Actin-GS-255B	No	0µM	96	65 (14)	66	79			
		10µM	104	73 (17)	77	90	7.762	12.17	7.9E-06
		100µM	102	75 (13)	76	90	9.541	10.79	6.3E-06
Beta1 RNAi x Actin-GS-255B	Yes	0µM	142	29 (23)	29	80			
		10µM	132	28 (22)	29	80	-1.000	0.000	0.623
		100µM	131	28 (23)	29	80	-1.425	0.000	0.561
Beta2 RNAi x Actin-GS-255B	No	0µM	99	66 (13)	67	77			
		10µM	101	75 (13)	75	104	9.974	8.919	2.2E-06
		100µM	102	75 (12)	75	103	10.17	9.091	2.0E-06
Beta2 RNAi x Actin-GS-255B	Yes	0µM	101	56 (15)	58	65			
		10µM	100	55 (16)	58	65	-1.131	0.000	0.595
		100µM	101	56 (16)	58	65	0.895	0.000	0.537

Female (Cohort 2)

Genotype	RU486	H_2O_2	N	Mean (SD)	Median	90%	Δ Mean %	Δ Median %	(p)
Beta1 RNAi x Actin-GS-255B	No	0µM	107	54 (16)	59	70			
		10µM	144	70 (18)	76	92	13.97	14.66	2.3E-07
		100µM	148	70 (17)	77	92	14.01	15.76	3.1E-07
Beta1 RNAi x Actin-GS-255B	Yes	0µM	141	61 (22)	62	93			
		10µM	120	60 (23)	62	93	-4.448	0.000	0.079
		100µM	119	59 (22)	62	93	-2.954	0.000	0.368
Beta2 RNAi x Actin-GS-255B	No	0µM	142	68 (15)	69	88			
		10µM	156	76 (15)	79	106	7.795	9.110	1.5E-05
		100µM	164	77 (15)	81	105	6.688	9.091	4.2E-05
Beta2 RNAi x Actin-GS-255B	Yes	0µM	80	55 (11)	59	68			
		10µM	79	55 (12)	59	68	0.314	0.000	0.7830
		100µM	79	55 (12)	59	68	0.175	0.000	0.7998

Male (Cohort 1)

Genotype	RU486	H_2O_2	N	Mean (SD)	Median	90%	Δ Mean %	Δ Median %	(p)
Beta1 RNAi x Actin-GS-255B	No	0µM	91	49 (19)	50	70			
		10µM	89	46 (19)	50	69	-0.613	0.000	0.619
		100µM	88	46 (19)	50	69	-0.620	0.000	0.608
Beta1 RNAi x Actin-GS-255B	Yes	0µM	78	45 (20)	46	67			
		10µM	80	44 (21)	45	64	-0.278	-0.616	0.336
		100µM	80	44 (22)	45	64	-0.323	-0.281	0.878
Beta2 RNAi x Actin-GS-255B	No	0µM	80	71 (13)	70	79			
		10µM	79	70 (12)	70	79	-0.765	0.000	0.7295
		100µM	79	70 (13)	70	79	-0.813	0.000	0.6968
Beta2 RNAi x Actin-GS-255B	Yes	0µM	100	53 (16)	58	70			
		10µM	101	48 (16)	49	68	-1.984	-10.12	0.5909
		100µM	100	47 (17)	47	68	-2.978	-11.11	0.2803

Male (Cohort 2)

Genotype	RU486	H_2O_2	N	Mean (SD)	Median	90%	Δ Mean %	Δ Median %	(p)
Beta1 RNAi x Actin-GS-255B	No	0µM	81	50 (15)	48	65			
		10µM	81	50 (16)	48	67	-0.247	0.000	0.965
		100µM	77	48 (15)	48	63	-1.744	0.000	0.606
Beta1 RNAi x Actin-GS-255B	Yes	0µM	78	43 (22)	47	64			
		10µM	80	42 (21)	46	64	-0.920	-0.837	0.900
		100µM	80	40 (22)	46	60	-2.444	-0.824	0.530
Beta2 RNAi x Actin-GS-255B	No	0µM	100	52 (15)	57	70			
		10µM	119	52 (15)	56	70	-0.846	0.152	0.7444
		100µM	119	53 (16)	58	70	0.171	0.334	0.9139
Beta2 RNAi x Actin-GS-255B	Yes	0µM	81	50 (16)	49	65			
		10µM	82	50 (16)	49	66	-0.420	0.000	0.9824
		100µM	80	53 (16)	57	66	1.547	7.127	0.4374

Supplementary Table S3. Lifespan statistical summary. Two cohorts of male and female progeny were transferred every other day onto fresh food and dead flies were recorded. Crosses in order: males x virgin females.

Cohort 1

Genotype	Sex	RU486	N	Mean (SD)	Median	90%	Δ Mean %	Δ Median %	(p)
w[1118] x Actin-GS-255B	F	No	92	66 (24)	64	92			
		Yes	99	67 (24)	66	92	3.08	5.55	0.0778
w[1118] x Actin-GS-255B	M	No	116	76 (23)	76	82			
		Yes	123	75 (23)	74	82	-2.11	-5.55	0.5618
β_1 RNAi x Actin-GS-255B	F	No	88	35 (24)	33	50			
		Yes	94	9.8(13)	10	11	-46.1	-43.7	3.11E-13
β_1 RNAi x Actin-GS-255B	M	No	111	84 (20)	85	99			
		Yes	113	34 (21)	33	42	-74.5	-78.6	0.0000
B_2 RNAi x Actin-GS-255B	F	No	93	85 (12)	84	96			
		Yes	93	32 (20	30	44	-51.1	-53.3	0.0000
β_2 RNAi x Actin-GS-255B	M	No	112	70 (23)	68	88			
		Yes	122	32 (10)	32	42	-67.9	-67.5	0.0000
CnC RNAi x Actin-GS-255B	F	No	102	67 (25)	68	89			
		Yes	82	58 (21)	57	74	-20.1	-23.3	2.30E-10
CnC RNAi x Actin-GS-255B	M	No	110	57 (21)	63	82			
		Yes	99	49 (18)	48	68	-16.4	-19.3	4.23E-07
Keap1 RNAi x Actin-GS-255B	F	No	97	61 (19)	61	80			
		Yes	83	75 (21)	75	92	14.3	16.3	0.0002
Keap1 RNAi x Actin-GS-255B	M	No	115	62 (21)	60	74			
		Yes	119	65 (23)	62	78	5.86	3.25	0.0390

Cohort 2

Genotype	Sex	RU486	N	Mean (SD)	Median	90%	Δ Mean %	Δ Median %	(p)
w[1118] x Actin-GS-255B	F	No	95	72 (21)	70	90			
		Yes	96	72 (21)	70	92	0.58	0.00	0.1875
w[1118] x Actin-GS-255B	M	No	95	76 (18)	76	80			
		Yes	88	75 (18)	72	78	-2.59	-7.69	0.2469
β_1 RNAi x Actin-GS-255B	F	No	110	11 (19)	10	12			
		Yes	99	41 (21)	42	49	-45.3	-44.2	1.51E-12
β_1 RNAi x Actin-GS-255B	M	No	105	78 (21)	76	98			
		Yes	112	37 (19)	39	42	-49.3	-46.7	4.09E-12
B_2 RNAi x Actin-GS-255B	F	No	96	89 (19)	87	100			
		Yes	94	29 (12)	29	43	-72.2	-70.0	0.0000
β_2 RNAi x Actin-GS-255B	M	No	101	75 (20)	74	80			
		Yes	103	34 (22)	33	40	-53.2	-50.0	0.0000
CnC RNAi x Actin-GS-255B	F	No	89	63 (21)	62	83			
		Yes	94	55 (18)	53	70	-19.1	-20.1	6.24E-10
CnC RNAi x Actin-GS-255B	M	No	112	63 (21)	62	83			
		Yes	119	56 (17)	56	77	-13.4	-14.1	0.0004
Keap1 RNAi x Actin-GS-255B	F	No	101	70 (19)	70	80			
		Yes	96	76 (20)	76	88	10.7	6.67	0.0013
Keap1 RNAi x Actin-GS-255B	M	No	123	61 (21)	61	71			
		Yes	112	64 (22)	65	79	3.78	4.25	0.0312

Supplementary Table S4. Hydrogen peroxide adaptation following loss of Keap1 in aged females and males. Flies were aged to 60 days either in the absence or presence of RU486. Crosses in order: males x virgin females.

Female

Genotype	RU486	H_2O_2	N	Mean (SD)	Median	90%	Δ Mean %	Δ Median %	(p)
Keap1 RNAi x Actin-GS-255B	No	0µM	112	34 (18)	36	46			
		10µM	118	35 (17)	36	46	1.67	0.000	0.5437
Keap1 RNAi x Actin-GS-255B	Yes	0µM	107	49 (20)	54	62			
		10µM	91	50 (18)	54	62	3.79	0.000	0.5821
Keap1 RNAi x Actin-GS-255B	No	0µM	185	25 (18)	26	50			
		10µM	196	26 (19)	26	50	1.35	0.000	0.5558
Keap1 RNAi x Actin-GS-255B	Yes	0µM	108	40 (19)	39	70			
		10µM	106	42 (19)	44	70	4.77	4.28	0.1413

Male

Genotype	RU486	H_2O_2	N	Mean (SD)	Median	90%	Δ Mean %	Δ Median %	(p)
Keap1 RNAi x Actin-GS-255B	No	0µM	118	24 (12)	28	46			
		10µM	123	26 (12)	28	46	2.76	2.55	0.4611
Keap1 RNAi x Actin-GS-255B	Yes	0µM	88	52 (18)	54	70			
		10µM	102	54 (19)	56	70	-2.63	-2.50	0.4396
Keap1 RNAi x Actin-GS-255B	No	0µM	132	27 (11)	26	48			
		10µM	123	27 (14)	28	49	-0.640	0.000	0.8549
Keap1 RNAi x Actin-GS-255B	Yes	0µM	119	56 (21)	57	72			
		10µM	117	55 (21)	57	72	-0.726	0.000	0.7931

Supplementary Table S5. Hydrogen peroxide adaptation in the presence or absence of RU486. Flies were aged to 60 days either in the absence or presence of RU486. Crosses in order: males x virgin females.

Female

Genotype	RU486	H_2O_2	N	Mean (SD)	Median	90%	Δ Mean %	Δ Median %	(p)
w[1118] x Actin-GS-255B	No	0µM	78	42 (18)	42	49			
		10µM	79	42 (18)	42	49	1.65	0.000	0.4889
w[1118] x Actin-GS-255B	Yes	0µM	86	44 (21)	44	52			
		10µM	76	46 (21)	46	52	3.50	1.50	0.1181
w[1118] x Actin-GS-255B	No	0µM	101	41 (20)	40	46			
		10µM	99	41 (20)	43	46	0.310	5.88	0.9281
w[1118] x Actin-GS-255B	Yes	0µM	103	40 (20)	43	66			
		10µM	100	41 (20)	43	66	0.622	0.000	0.7242

Male

Genotype	RU486	H_2O_2	N	Mean (SD)	Median	90%	Δ Mean %	Δ Median %	(p)
w[1118] x Actin-GS-255B	No	0µM	61	35 (21)	34	40			
		10µM	68	33 (22)	34	40	-4.01	0.000	0.2806
w[1118] x Actin-GS-255B	Yes	0µM	64	37 (18)	37	42			
		10µM	74	36 (19)	36	41	-2.21	-1.11	0.2148
w[1118] x Actin-GS-255B	No	0µM	79	30 (20)	30	46			
		10µM	80	32 (20)	30	46	2.68	0.000	0.4316
w[1118] x Actin-GS-255B	Yes	0µM	66	32 (14)	34	44			
		10µM	78	32 (14)	34	44	0.083	0.000	0.9676
